# Gut Microbial Alterations in Diarrheal Baer's Pochards (*Aythya baeri*)

**DOI:** 10.3389/fvets.2021.756486

**Published:** 2021-10-14

**Authors:** Li Xi, Xinxi Qin, Yumin Song, Jincheng Han, Zhiqiang Li, Jinliang Zhang

**Affiliations:** ^1^Department of Animal Science, College of Biology and Food, Shangqiu Normal University, Shangqiu, China; ^2^Henan Engineering Research Center of Development and Application of Green Feed Additives, College of Biology and Food, Shangqiu Normal University, Shangqiu, China; ^3^Linyi Agricultural Science and Technology Career Academy, Linyi, China

**Keywords:** Baer's pochard, gut microbiota, ITS, composition, diarrhea

## Abstract

The structure and composition of gut microbiota correlate with the occurrence and development of host health and disease. Diarrhea can cause alterations in gut microbiota in animals, and the changes in the gut microbial structure and composition may affect the development of diarrhea. However, there is a scarcity of information on the effects of diarrhea on gut fungal composition and structure, particularly in Baer's pochard (*Aythya baeri*). The current study was performed for high-throughput sequencing of the fungal-specific internal transcribed spacer 1 (ITS-1) to detect the differences of gut mycobiota in healthy and diarrheal Baer's pochard. Results showed that the gut mycobiota not only decreased significantly in diversity but also in structure and composition. Statistical analysis between two groups revealed a significant decrease in the abundance of phylum Rozellomycota, Zoopagomycota, Mortierellomycota, and Kickxellomycota in diarrheal Baer's pochard. At the genus levels, fungal relative abundance changed significantly in 95 genera, with 56 fungal genera, such as *Wickerhamomyces, Alternaria, Penicillium, Cystofilobasidium*, and *Filobasidium*, increasing significantly in the gut of the diarrheal Baer's pochard. In conclusion, the current study revealed the discrepancy in the gut fungal diversity and community composition between the healthy and diarrheal Baer's pochard, laying the basis for elucidating the relationship between diarrhea and the gut mycobiota in Baer's pochard.

## Introduction

The gut microbiota is a group of microorganisms that maintains the balance of the intestinal microecological system in birds. They participate in the nutrition, metabolism, immunity, and development of the host (1, 2). Bacteria, fungus, and protozoa inhabiting the gut may engage in a commensal, symbiotic, or parasitic relationship, resulting in a stable intestinal environment (3). Gut microbial alterations can cause many diseases, such as diabetes, obesity, and diarrhea (4, 5).

Although the proportion of fungi in gut microbiota is low, it is closely associated with the feeding, immunity, and metabolic health of the host (6–8). Studies on the relationship between gut fungi and host health have confirmed that an imbalance in the gut fungal flora can cause diarrhea (9, 10). The intestinal abundance of Neocallimastigomycota in diarrhea yaks was found to be significantly higher than that of healthy yaks, suggesting that Neocallimastigomycota may be associated with diarrhea (11). Fecal fungi showed significant correlations with irritable bowel syndrome (IBS) symptoms (12). Moreover, bacterial–fungal interactions markedly declined in diarrhea-predominant IBS patients (13). *Candida glabrata* and *Candida krusei* were identified from Amazon parrot, dove, macaw, and cockatiel with gastrointestinal disease (14). Recent studies have shown that *Candida* species is often a conditional pathogen that has the opportunity to play a pathogenic role when the host's gut microecological balance is disrupted (15, 16). *Aspergillus fumigatus* is a widespread opportunistic pathogenic fungus in the natural environment and can cause severe pulmonary aspergillosis and other diseases. For instance, *Aspergillus fumigatus* could cause multisystemic damage to the ostrich body, showing diarrhea, vomiting, severe respiratory suppression, and even death (17). Given the importance of the gut fungal community in dysbacteriosis associated with diarrhea and inflammatory bowel disease, it is crucial to learn more about its composition and structure.

Baer's pochard (*Aythya baeri*) has been listed in the Category I of the State Key Protected Wildlife List in China and the CR/EN Category of the International Union for Conservation of Nature (IUCN) (18). As a wild bird, it is not easy to get timely diagnosis and treatment after diarrhea, which may result in death and threaten the continuity of the species. Currently, most of the studies on gut microecology in birds focus on gut bacterial diversity. High-throughput sequencing is less used to investigate the gut fungal diversity of birds, especially Baer's pochards. 18S rDNA, and ITS-based gene sequencing technologies have been widely used in fungal taxonomic identification (19–21). The in-depth analysis of changes in the structure and composition of gut mycobiota in diseased animals is beneficial to reveal the correlation of disease and flora, identify new pathogens, establish flora beneficial to maintaining the healthy growth of the body, and put forward effective prevention and control measures (22, 23). In this study, high-throughput sequencing technology was used to study the gut fungal composition and structure in the healthy and diarrheal Baer's pochards in order to investigate the effect of diarrhea on the gut fungal diversity of the Baer's pochards. Our study could provide molecular data for further protection of this endangered water bird and lay a scientific theoretical basis for population recovery and the formulation of effective protection policies and measures.

## Materials and Methods

### Sample Collection

Fresh fecal samples were collected from eight healthy adult Baer's pochards (group C; marked as C1–C8) and eight diarrheal adult Baer's pochards (group D; marked as D1–D8) from the National wetland park (Shangqiu, China). The samples in each group were collected immediately after Baer's pochard defecation to avoid contamination by environmental microorganisms. For further examination, the feces were put in sterile tubes, snap frozen, and stored at −80°C.

### Internal Transcribed Spacer Amplification and Sequencing

The feces were processed as previously described (11). The fungal genomic DNA of fecal samples was extracted using Rapid Fungi Genomic DNA Isolation Kit (Sangon Biotech, Shanghai, China) according to the instructions of the manufacturer. The DNA sample was purified and quantified by 0.8% agarose gel electrophoresis and was diluted with sterile water to 1 ng/μl. Using the diluted genome DNA as the template, PCR amplification was performed using fungal ITS gene primers (ITS1F: 5′-CTTGGTCATTTAGAGGAAGTAA-3′ and ITS2R: 5′-GCTGCGTTCTTCATCG ATGC-3′), and Phusion High-Fidelity PCR Master Mix with GC Buffer (Thermo Fisher Scientific, Waltham, USA). The PCR products were analyzed using 2% agarose gel electrophoresis, and the equivalent PCR products were mixed according to the concentration. The sequencing library was prepared by Illumina TruSeq Nano DNA LT Library Prep Kit and then underwent quality inspection and quantitative analysis. The qualified library with concentrations above 2 nmol/L and single peak were combined and performed 2 × 250-bp paired-end sequencing on Illumina HiSeq 2500 platform (Illumina, CA, USA).

### Bioinformatics and Statistical Analysis

To ensure the accuracy of the obtained sequences, raw reads were first filtered by Trimmomatic v0.33 software (24), and then the Cutadapt 1.9.1 software (25) was used to obtain high-quality reads without the primer sequence. Subsequently, the above high-quality reads were spliced through the FLASH v1.2.7 software (26) to obtain the clean reads. Finally, the UCHIME v4.2 software (27) was used to identify and remove the chimera sequence in clean reads to obtain effective reads. The effective reads were clustered into operational taxonomic unit (OTU) using VSEARCH algorithm (1.9.6) (28) according to the UNITE ITS database at 97% sequence similarity. The characteristic sequences are taxonomically annotated using a naive Bayesian classifier. Abundance tables at different classification levels were generated using QIIME 2 software (29). Community structure maps at each taxonomic level of the sample were drawn using the R v3.0.3 software (30). Rarefaction curves and rank abundance curves evaluating the sequencing depth and the species richness and evenness contained in the samples were calculated by the R v3.0.3 software. The α and β diversity indexes were calculated using QIIME 2 software. PCoA (31) were illustrated using R v3.0.3 software to characterize the fungal diversity among samples. Line discriminant analysis (LDA) effect size (LEfSe) (32) was used to find biomarkers with statistical differences between groups. *T*-testing of species abundance data between groups was performed using the Metastats software (33), and species with significant differences were screened based on *p*-values. A *p*-value < 0.05 was considered statistically significant.

## Results

### Sequencing Quality Assessment

**Figure 1:**
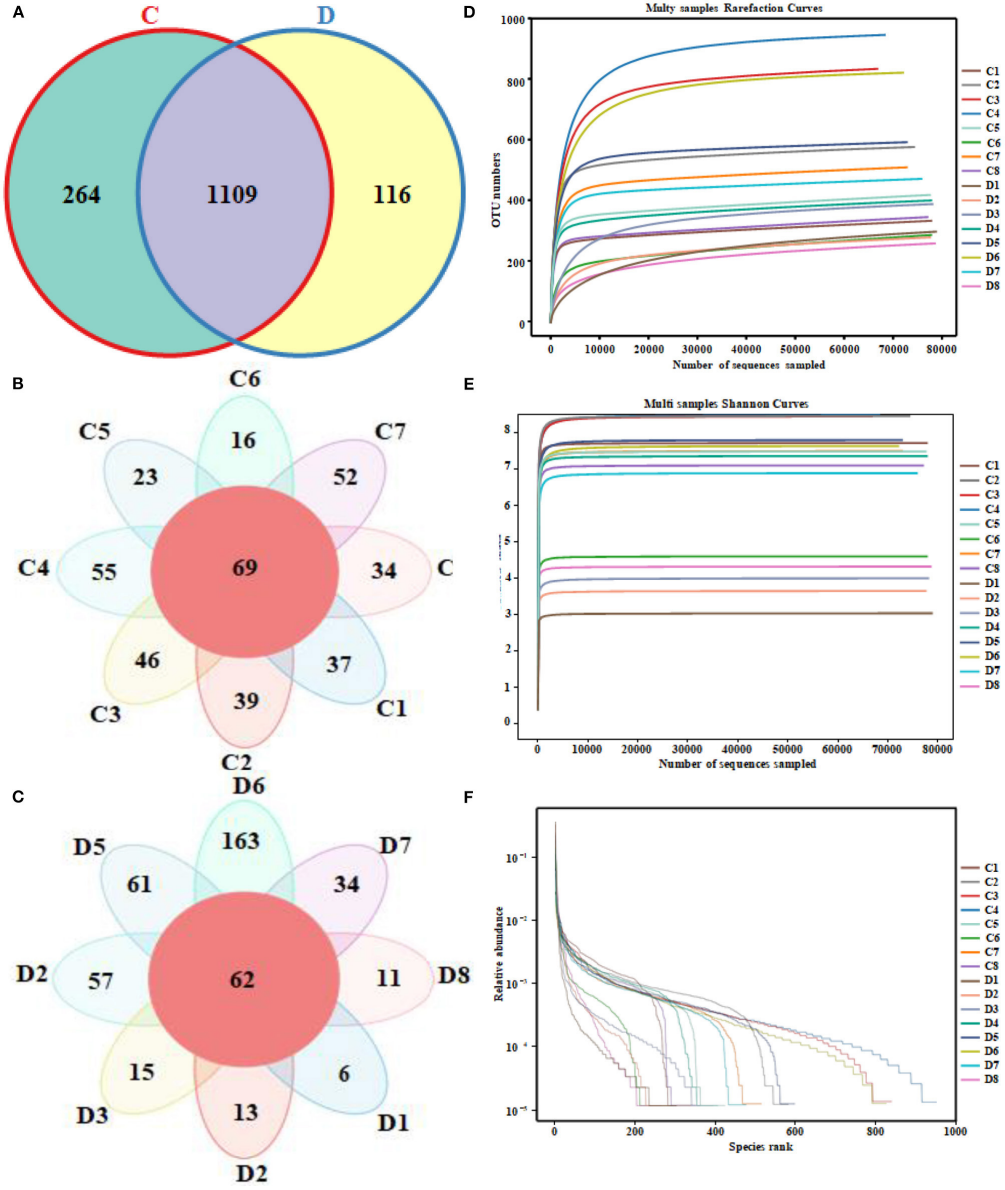
Venn diagrams and sequencing quality evaluation. **(A)** Venn diagram of operational taxonomic units (OTUs) distribution between group C and group D. **(B,C)** Flower diagrams of the OTU shared among the samples within the group C and group D, respectively. **(D)** Rarefaction curve of 16 fecal samples in both groups. **(E)** Shannon index curve of 16 fecal samples in both groups. **(F)** Rank abundance curve of 16 fecal samples in both groups.

A total of 1,280,719 (*C* = 639,738, *D* = 640,981) original sequences were obtained in the 16 fecal samples (Table 1). The effective reads [1,266,771 (*C* = 632,515, *D* = 634,256)] were obtained after removing the low-quality data, with an average of 79,173 effective reads per sample. The obtained effective reads were clustered using VSEARCH 1.9.6 software with 97% consistency, yielding 1,489 OTUs (*C* = 1,373, *D* = 1,225). Notably, there were 264 OTUs in the healthy group that were not detected in the diarrheal group, while 116 OTUs were present only in the diarrheal group (Figure 1A). The healthy samples shared 69 OTUs, while the diarrheal samples shared 62 OTUs (Figures 1B,C). The rarefaction curve was used to judge whether the sequencing data volume reflects the species diversity in the sample. When the sequencing amount reaches 10,000, all of the curves tend to be flat, indicating that the amount of sampling is reasonable, the sequencing depth has been substantially covering all the species in the sample, and can well reflect the fungal community structure and diversity of all the samples (Figures 1D,E). Except for the smooth decline in the rank abundance curves for C3, C4, and D6 showing better abundance and evenness, rapid and steep decline in other samples indicating that these samples had lower fungal diversity and a high proportion of dominant fungi (Figure 1F).

**Table 1:** The sequencing data processing results of fecal samples.

**Sample ID**	**Raw reads**	**Clean reads**	**Effective reads**	**AvgLen (bp)**	**Effective (%)**
C1	79,788	79,127	78,976	244	98.98
C2	79,939	79,265	79,117	242	98.97
C3	80,016	79,246	78,989	243	98.72
C4	79,958	79,208	78,823	246	98.58
C5	80,125	79,447	79,303	245	98.97
C6	80,098	79,491	79,424	242	99.16
C7	80,031	79,338	78,992	243	98.70
C8	79,783	79,091	78,891	237	98.88
D1	80,208	79,586	79,443	246	99.05
D2	80,232	79,595	79,156	244	98.66
D3	79,871	79,343	79,241	226	99.21
D4	80,272	79,584	79,352	238	98.85
D5	80,063	79,368	79,093	241	98.79
D6	79,996	79,309	79,020	240	98.78
D7	80,043	79,352	79,331	241	99.11
D8	80,296	79,706	79,620	239	99.16

### Effects of Diarrhea on Gut Fungal Diversity

**Figure 2:**
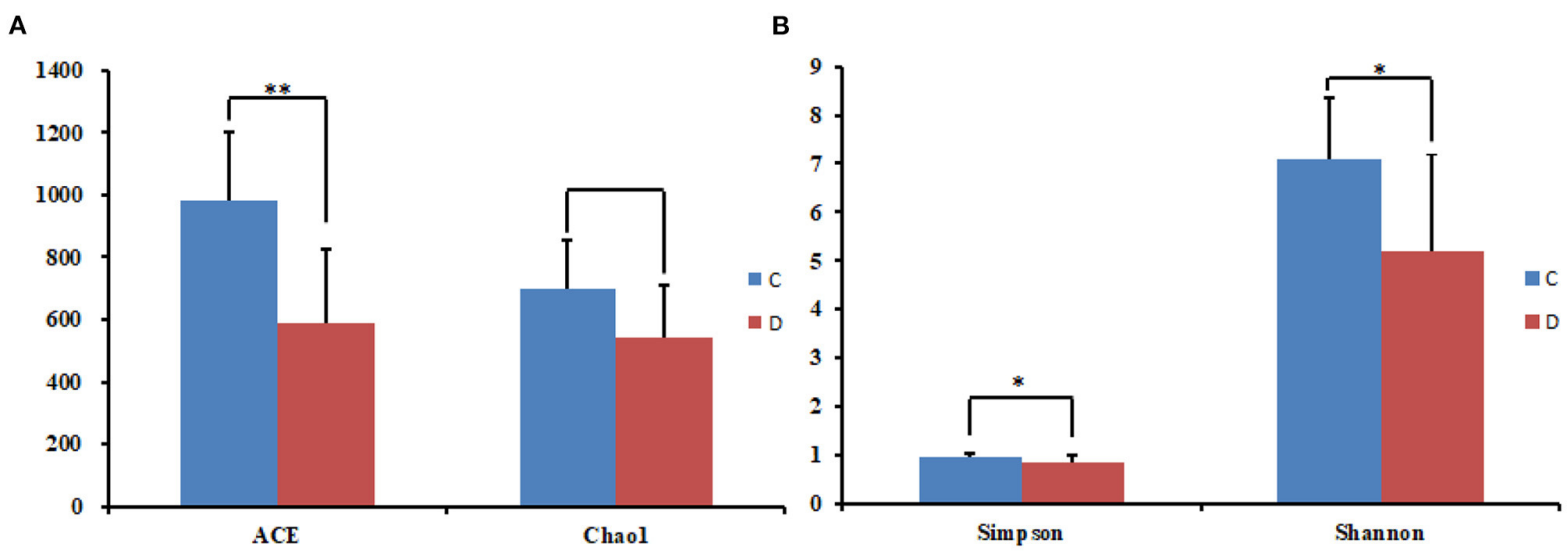
Statistical results of the alpha diversity between the two groups. **(A)** ACE and Chao1 index; **(B)** Simpson and Shannon index. *p < 0.05, **p < 0.01.

**Figure 3:**
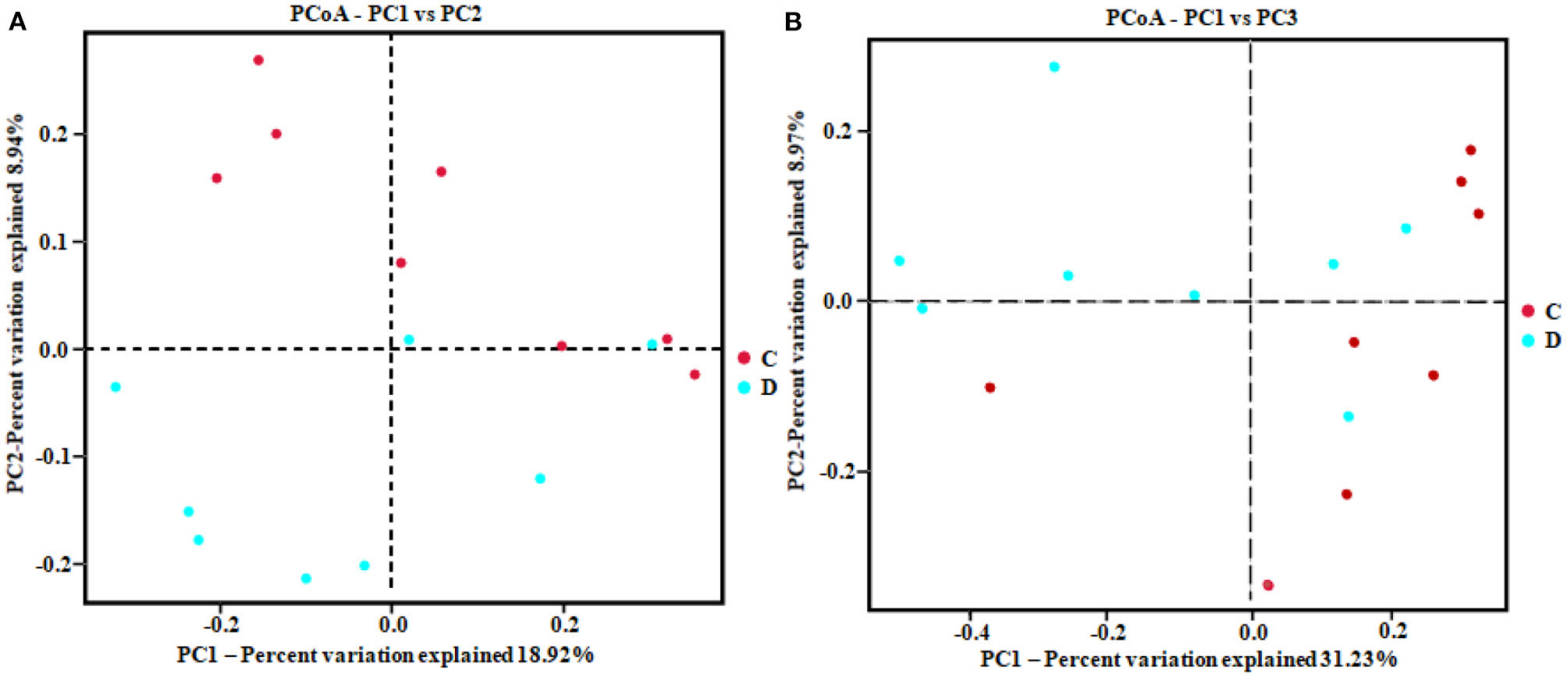
Principal coordinates analysis (PCoA) diagram of heterogeneity between the two groups. Scatterplot from PCoA based on binary-Jaccard **(A)** and Bray–Curtis dissimilarity **(B)** in gut fungal community.

The alpha diversity index of the samples was evaluated using the QIIME2 software. The Good's coverage values for all samples ranged from 99.90 to 99.95%, showing high library coverage. The average Chao1 index was 699.84 and 539.47 in the healthy and diarrheal groups, respectively, with no significant difference (*p* > 0.05). ACE indices in the healthy and diarrheal group were 982.29 and 586.18 (*p* < 0.01), respectively. The Shannon index was 7.09 in the healthy group, 5.21 in the diarrheal group, with a significant difference (*p* < 0.05). The Simpson index also varied significantly (*p* < 0.05) between the two groups, with 0.97 in the healthy group and 0.85 in the diarrheal group. Alpha diversity index statistics showed that gut fungal diversity in diarrheal Baer's pochard was significantly lower in the healthy group (Figure 2). Principal coordinates analysis (PCoA) further demonstrated beta diversity differences between the two groups. PCoA based on the binary-Jaccard and Bray–Curtis distance revealed that the principal compositions of gut fungal diversity in the control group differed from that in diarrheal groups due to diarrheal effect (Figures 3A,B).

### Effect of Diarrhea on the Gut Fungal Structure and Composition

**Figure 4:**
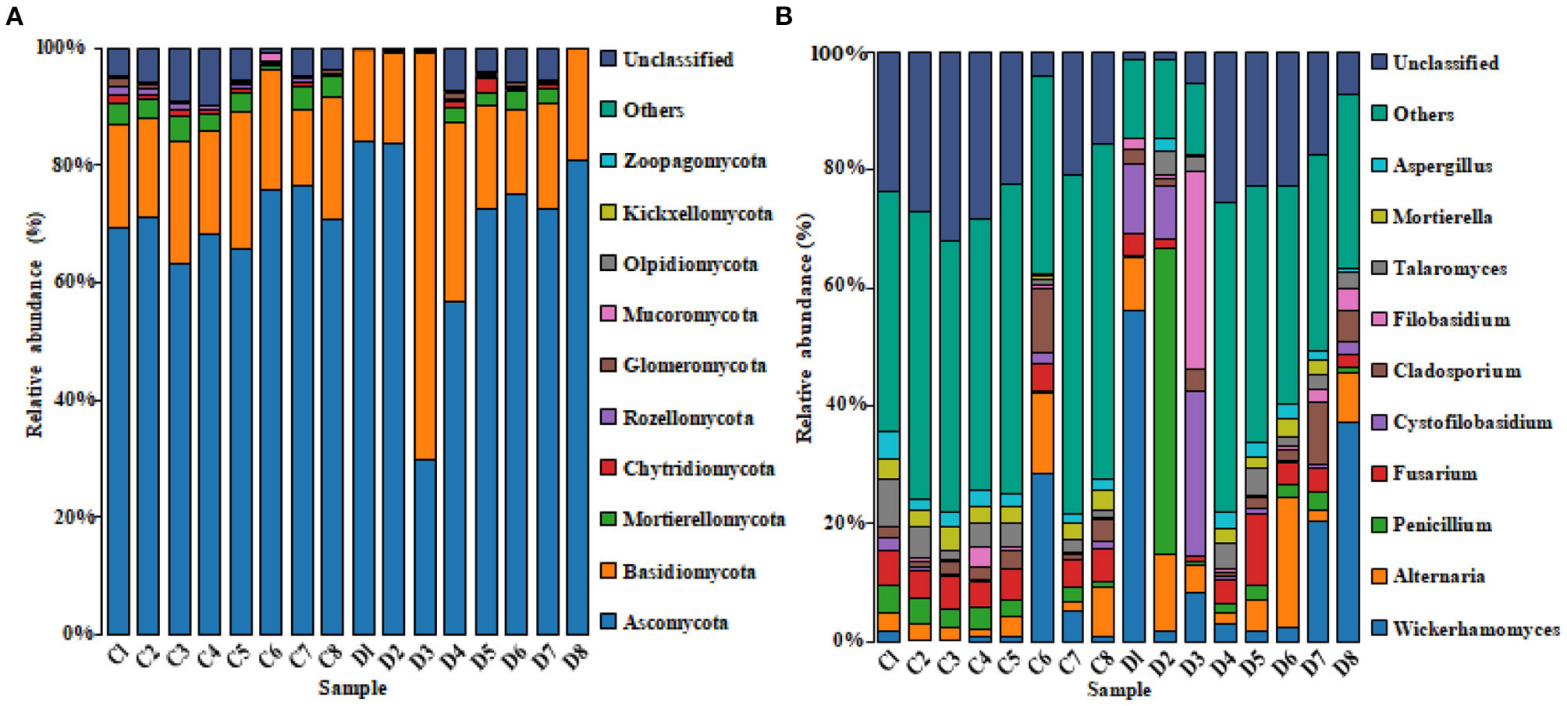
The histogram of the top 10 species distribution at phylum **(A)** and genus **(B)** levels.

**Figure 5:**
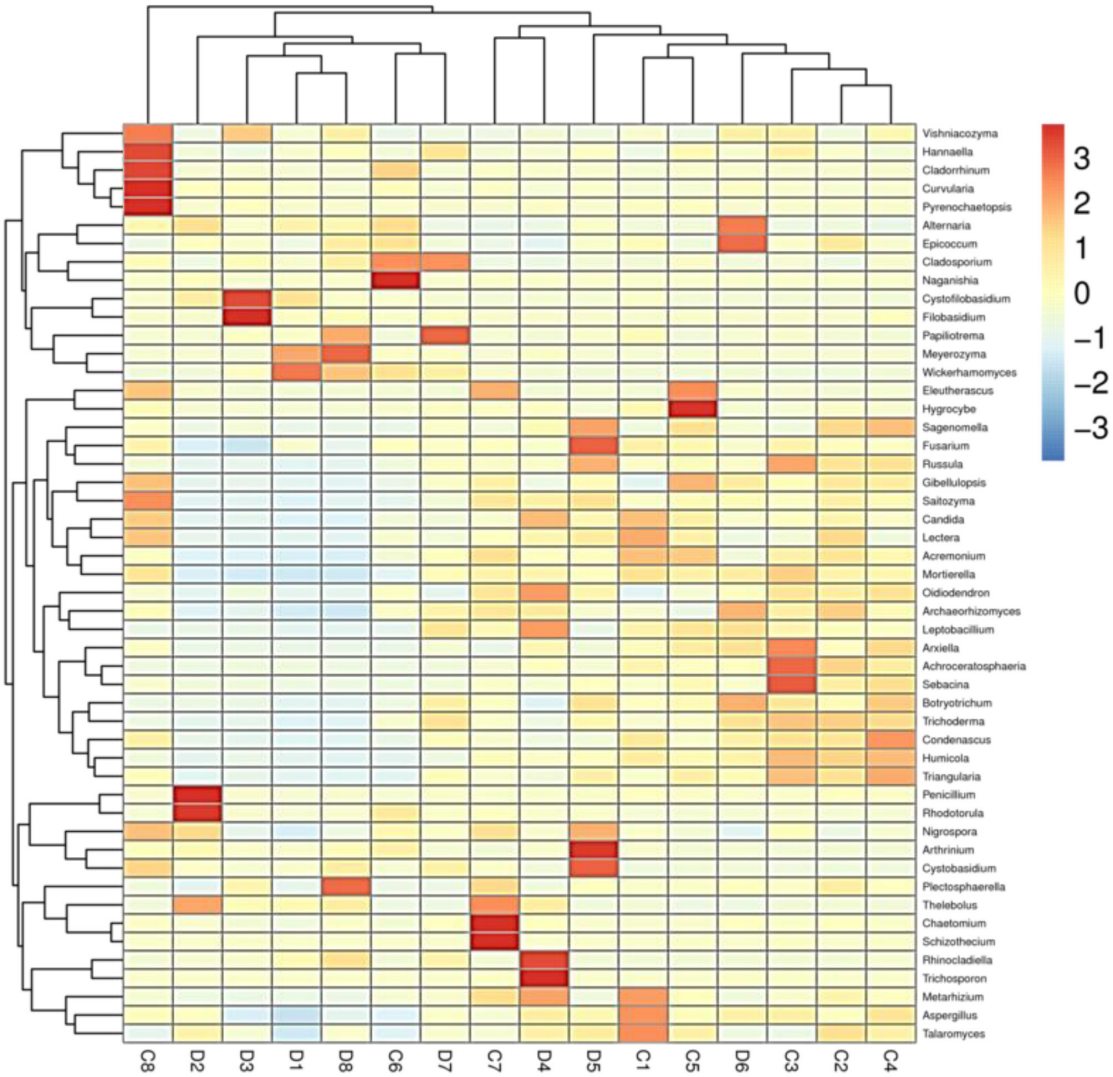
Heatmap at genus level of each sample (top 50).

The histogram of species distribution showed relative abundances of dominant fungal taxa in all samples. In the current study, 10 phyla and 388 genera were identified from all samples, and the top 10 phyla and genera rank in abundance is presented in Figure 4. At phylum level, Ascomycota (70.26% in group C, 69.47% in group D) and Basidiomycota (18.88% in group C, 25.15% in group D) were the dominant fungal phyla in all samples, accounting for 89.14 and 94.62% of the taxonomic groups identified, respectively (Figure 4A). The other five phyla with lower abundance, including Mortierellomycota (3.14% in group C, 1.35% in group D), Chytridiomycota (0.63% in group C, 0.54% in group D), Rozellomycota (0.77% in group C, 0.23% in group D), Glomeromycota (0.54% in group C, 0.28% in group D), and Mucoromycota (0.27% in group C, 0.09% in group D) accounted for 5.35 and 2.49% of all fungal taxa in two groups, respectively. At the genus level, the relative abundances of *Wickerhamomyces, Alternaria, Penicillium, Cystofilobasidium*, and *Filobasidium* were observed higher in the diarrheal group (4.93, 4.67, 2.75, 0.85, and 0.78% in group C vs. 16.68, 8.15, 8.00, 6.83, and 5.53% in group D). Conversely, the relative abundances of *Fusarium, Talaromyces*, and *Mortierell*a in the diarrheal group decreased (5.15, 3.43, and 2.79% in group C vs. 3.95, 2.72, and 1.30%). The cluster heatmap of species abundance at genus level is shown in Figure 5.

**Figure 6:**
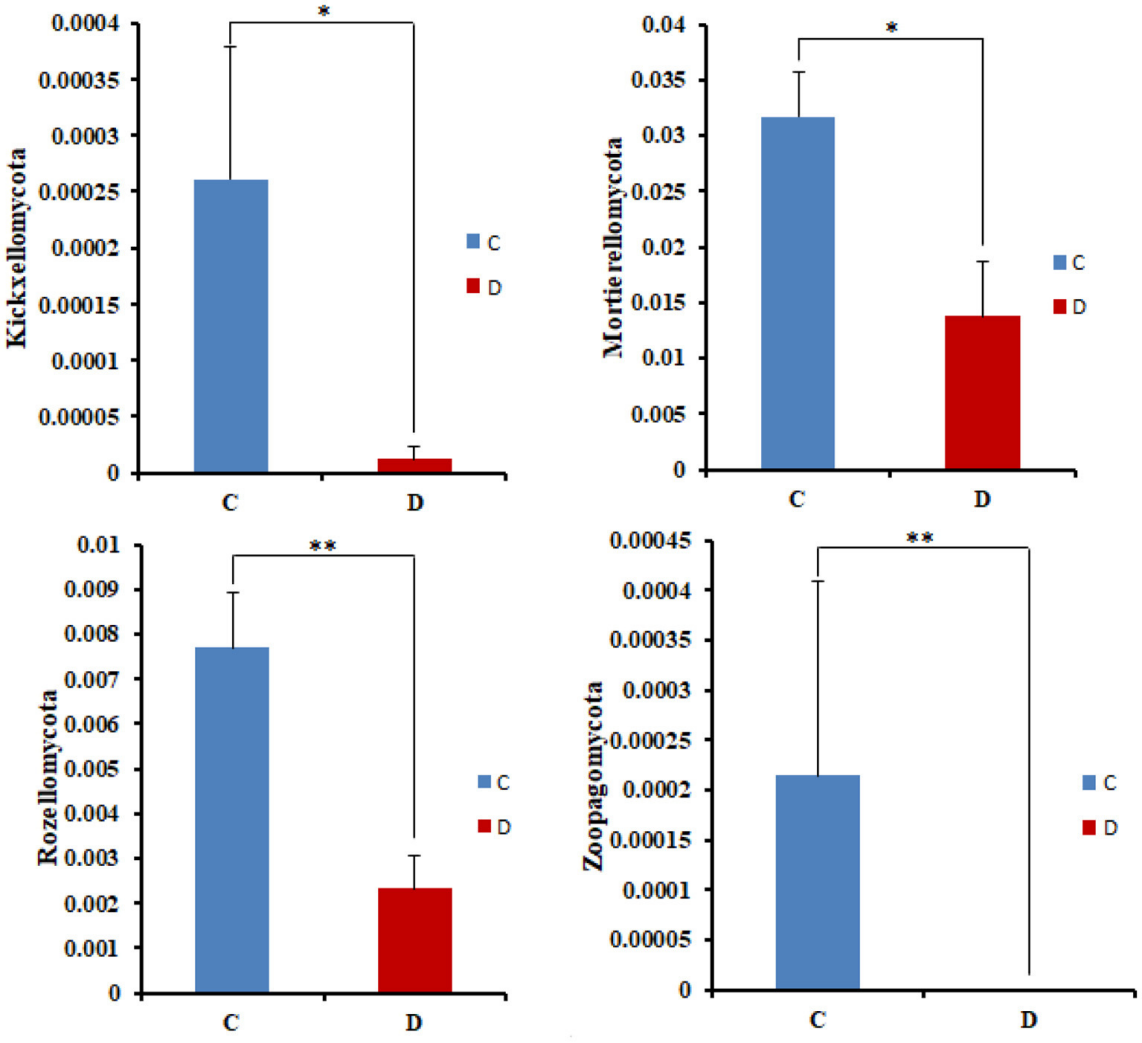
Comparison with the fungal community structure at phylum level in the two groups. *p < 0.05, **p < 0.01.

**Figure 7:**
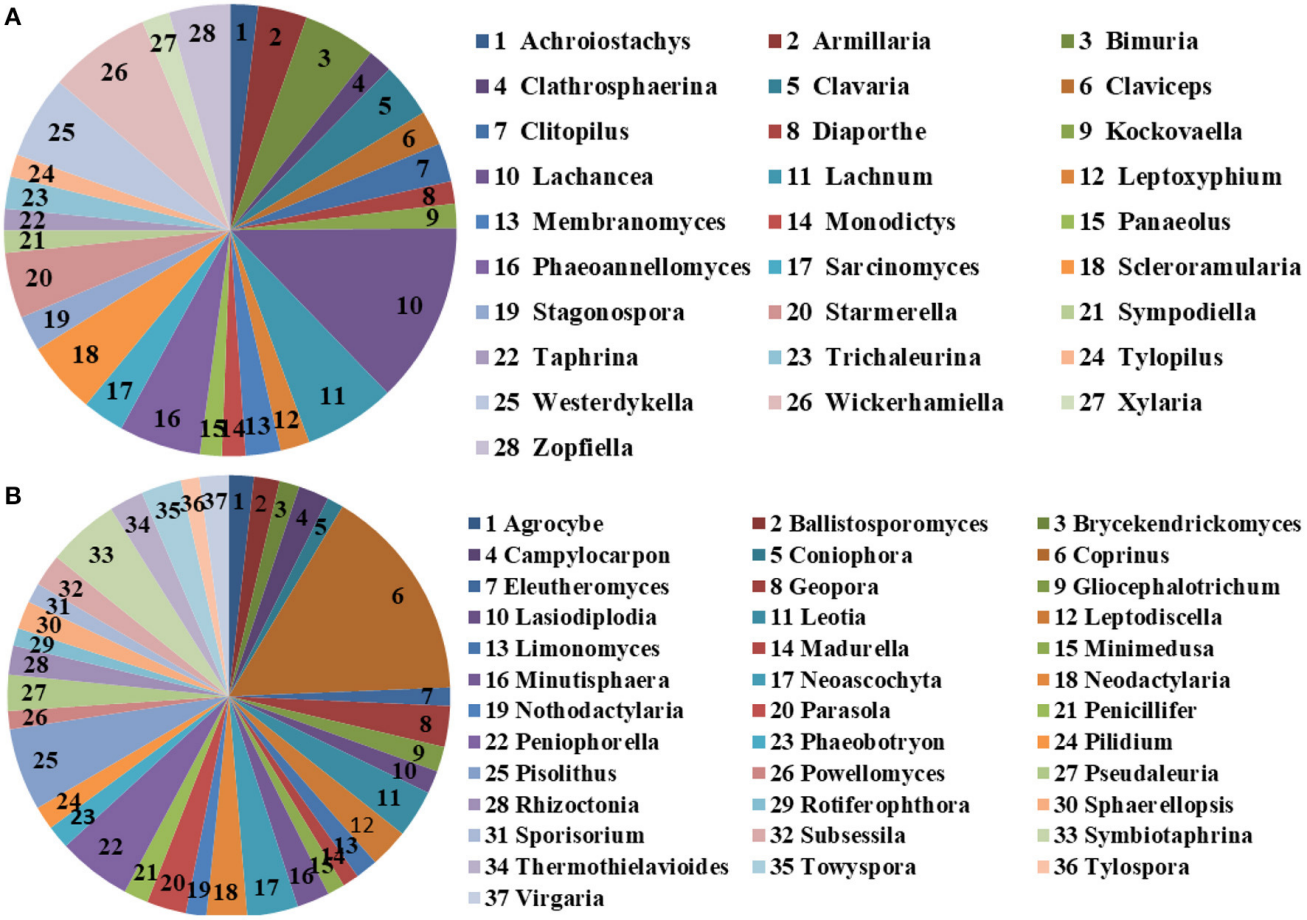
The unique fungal genera of the two groups. **(A)** Genera detected only in the diarrheal group. **(B)** Genera detected only in the healthy group. The sector area represents the abundance of these genera.

**Figure 8:**
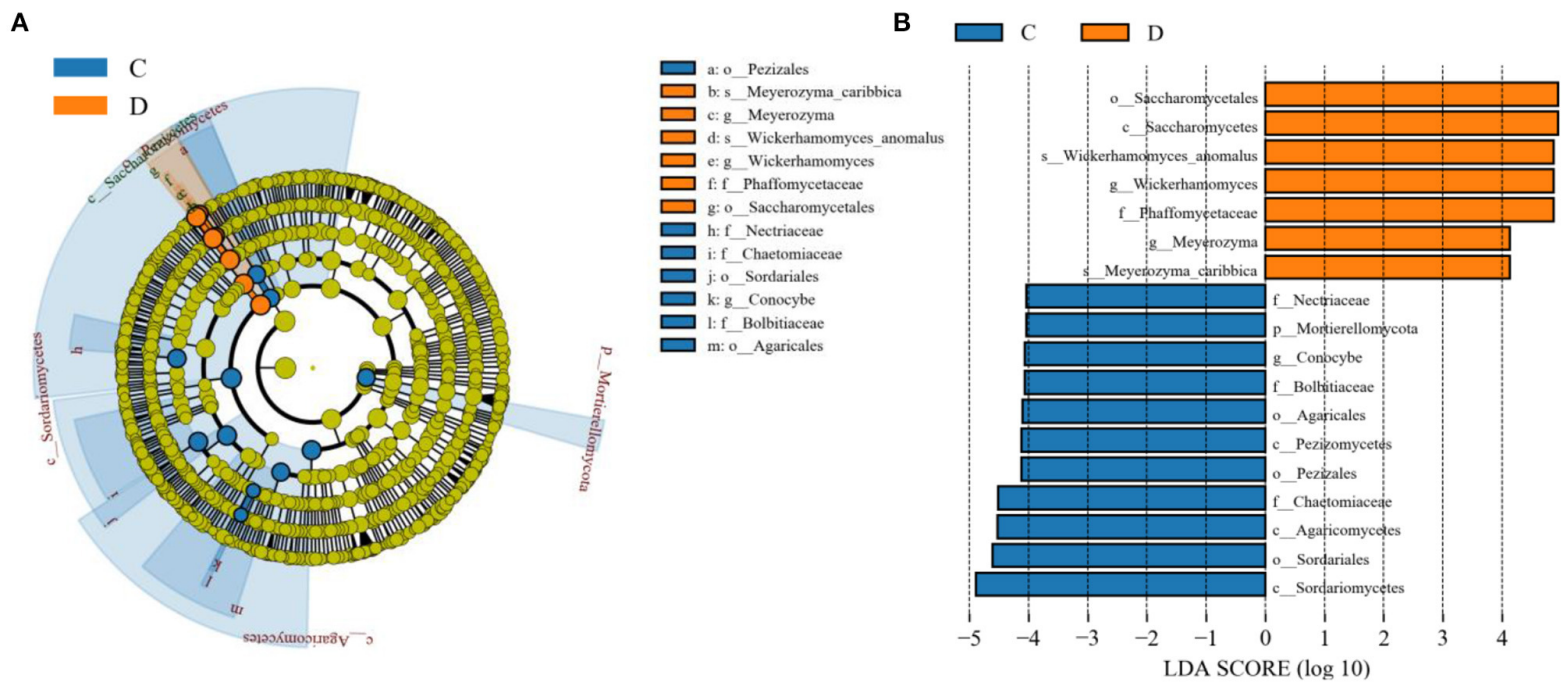
Effect size (LEfSe) analysis of intergroup samples. **(A)** LEfSe cladogram of fungal communities between the two groups. **(B)** Distribution histogram based on line discriminant analysis (LDA) value (LDA scores > 4).

The microbial community significant differences between the two groups were analyzed by Metastats software. At the phylum level, the richness of Rozellomycota, Zoopagomycota, Mortierellomycota, and Kickxellomycota in the diarrheal group significantly decreased than the healthy group (*p* < 0.05; Figure 6). Furthermore, of the 95 fungal genera with significant differences, the relative abundance of 56 fungal genera increased significantly in the diarrheal group. In comparison, the relative abundance of the remaining 39 fungal genera decreased significantly (Supplementary Table 1). Notably, 28 fungal genera were detected only in the diarrhea group, while 37 fungal genera in the healthy group disappeared in the diarrhea group, showing a significant diarrhea correlation (Figure 7). LEfSe was analyzed for significant differences between healthy and diarrheal groups. One order (Saccharomycetales), one family (Phaffomycetaceae), and two genera (*Wickerhamomyces* and *Meyerozyma*) were significantly more abundant in the diarrheal group. The relative abundances of three orders (Agaricales, Pezizales, and Sordariales) and three families (Nectriaceae, Bolbitiaceae, and Chaetomiaceae) were significantly higher in the healthy group than in the diarrheal group (Figures 8A,B).

### Correlation Network Analysis

**Figure 9:**
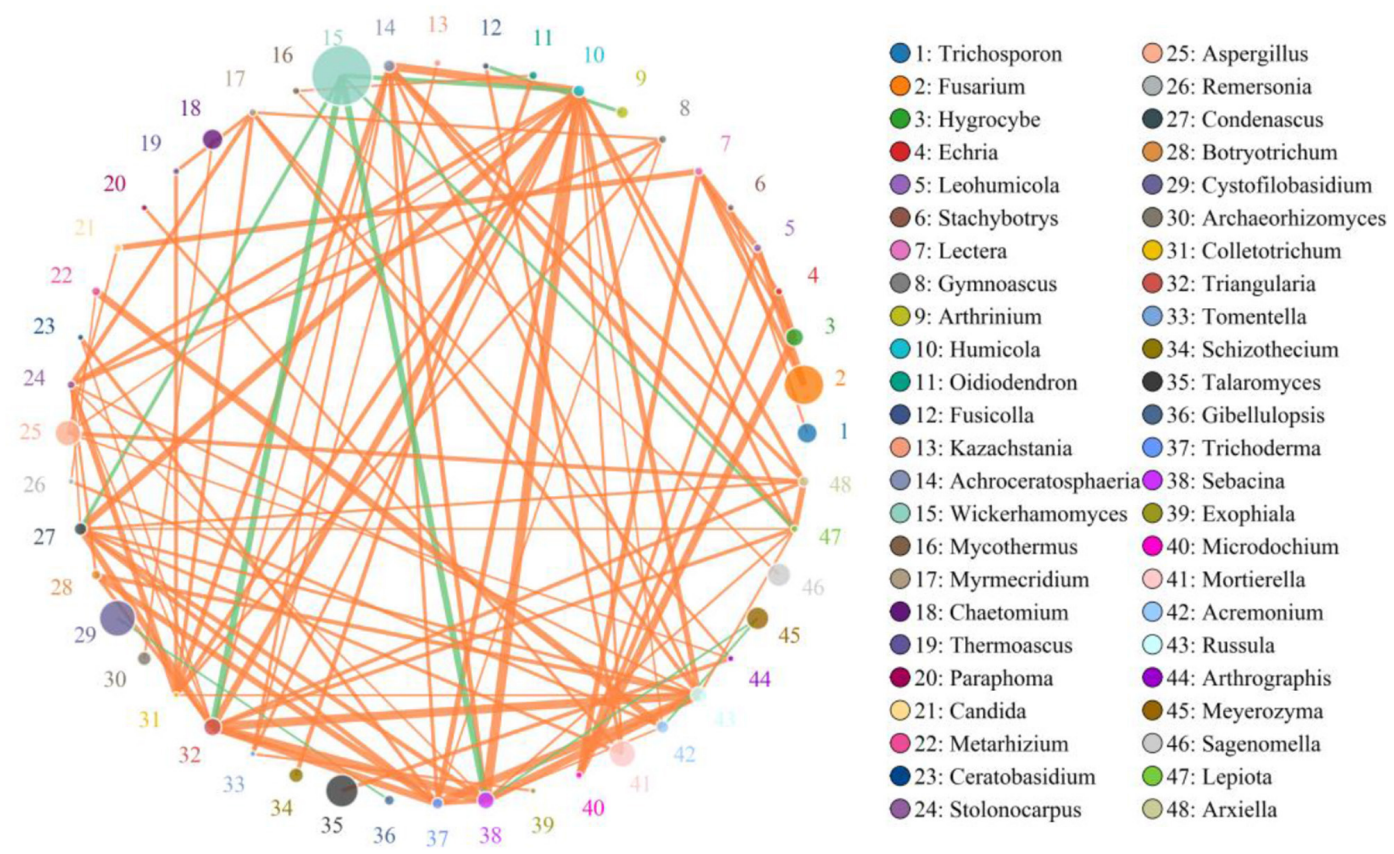
Network diagram of fungi at the genus level in the gut of Baer's pochard. The circles with different colors represent different genera, and the circle size represents the average abundance of each genera. The thickness of the line between two circles represents the strength of the correlation. The orange line represents the positive correlation, while the green line represents the negative correlation.

A species correlation network with 937 edges and 78 nodes was drawn based on the python programming language. The top 48 genera with the highest correlation are shown in Figure 9. *Wickerhamomyces* was negatively associated with *Triangularia* (0.8794), *Sebacina* (0.8794), *Humicola* (0.8294), *Condenascus* (0.8147), and *Lepiota* (0.7994), positively associated with *Meyerozyma* (0.6941) and *Rhinocladiella* (0.5451). *Alternaria* was positively related to *Cystofilobasidium* (0.5059), *Arthrinium* (0.6284), and *Curvularia* (0.5636). *Penicillium* was positively associated with *Talaromyces* (0.6471), *Aspergillus* (0.6265), *Triangularia* (0.5941), *Sebacina* (0.6324), *Russula* (0.6029), *Condenascus* (0.6324), *Acremonium* (0.5971), *Achroceratosphaeria* (0.5206), *Humicola* (0.6441), *Trichoderma* (0.5588), and *Botryotrichum* (0.5235). *Cystofilobasidium* was positively associated with *Arthrinium* (0.6431). *Filobasidium* was positively associated with *Meyerozyma* (0.5647) and *Rhinocladiella* (0.6377) (Supplementary Table 2).

## Discussion

Baer's pochard is one of the most endangered birds in the world (18). The International Union for Conservation of Nature created the International Single Species Action Plan (ISSAP) for Baer's pochard in 2014. China has an important distribution of Baer's pochard, with a population of about 600–800, which plays a key role in protecting Baer's pochard world population. The National wetland park (Shangqiu, China) is an important wintering ground and resting place for migratory birds. Baer's pochard was first observed in the wetland park in 2017. The largest population was over 150 observed in winter 2018, representing 15% of the global population. The wetland park was designated as “an important breeding site for Baer's pochard” in September 2019, indicating that it is essential for keeping birds in the park, and critical for the survival of the population. The health and breeding of Baer's pochard in these breeding sites are of great significance for population recovery and expansion.

A variety of pathogens such as bacteria, viruses, and fungi can cause animal diarrhea-related diseases, leading to animal nutrition absorption disorders, vomiting, diarrhea, dehydration, and other clinical symptoms (34). Wildlife is more vulnerable to the threat of diarrhea due to the lack of timely, effective veterinary intervention. Studies have confirmed that the gut microbiome plays an important role in host growth, development, and immune response (35, 36). Changes in the gut microbial community not only lead to gastrointestinal diseases, such as metabolic syndrome, obesity, and diarrhea (37, 38), but also cause diseases outside the digestive system, including central nervous system disorders (39), cardiovascular disease (40), etc. The gut microbial imbalance may also result in functional diarrhea (41, 42). Beneficial microbes can form a biological barrier by competing for intestinal mucosa cells, preventing pathogenic and conditional pathogenic microbes from invading. When the balance of gut microbiota is broken, the pathogenic microbes and their toxins increase relatively, which will damage the intestinal mucosa barrier, increase its permeability. Intestinal pathogenic microbes and their antigens can activate mast cells, and the sensitized mast cells can release histamine, serotonin, prostaglandin, trypsin, and other active substances to enhance smooth muscle contraction, increase intestinal peristalsis, and eventually result in diarrhea (43, 44). Moreover, long-term diarrhea can further aggravate gut microbial dysbiosis and diarrhea. Therefore, the investigation of the composition and structure of gut microbiota is conducive to mastering the pathogenesis of diarrhea and formulating effective prevention and treatment measures. The analysis of gut microbiota, which is closely related to the occurrence and development of diseases, mainly focused on bacteria. The research on fungi, especially the gut fungal alterations of wild protected animals under different health states, has rarely been reported.

The main fungal genera known to cause diarrhea are *Candida, Eurotium, Cryptococcus, Mucor, Histoplasma*, and *Fusarium* (13, 14, 45–48). Moreover, infection with other pathogenic microorganisms, abuse of antibiotics, or reduced immune function can disrupt the inter-constraints between normal gut resident microorganisms, resulting in fungus-associated diarrhea (49, 50). Given the importance of the gut fungal community in dysbacteriosis associated diarrhea, it is crucial to learn more about its composition and structure. In the current study, we systematically investigated the gut fungal alterations in Baer's pochard associated with diarrhea and explored the effect of diarrhea on the gut fungal diversity of the Baer's pochards. Results showed that diarrhea significantly decreased the gut fungal diversity and altered the fungal community composition and structure of Baer's pochard. LEfSe and Metastats analyses between healthy and diarrheal groups found significant differences in fungi from phyla to species that may be closely related to the health status of Baer's pochard. Our study could provide molecular data for further protection of this endangered water bird, and provide a scientific theoretical basis for population recovery and the formulation of effective protection policies and measures.

This study demonstrated that Ascomycota, Basidiomycota, and Mortierellomycota were dominant phyla in the gut of healthy and diarrheal Baer's pochard. The above phyla were also found to be preponderant in the gut mycobiota of ghost moth, silkworm, human, dog, and cattle (51–56). Among lower abundance representatives of fungi, Mortierellomycota was found with a higher abundance in healthy soil (57). Rozellomycota and Chytridiomycota dominate the aquatic fungal community, and many of these could be parasitic within the cells of fungi, plants, or animals, especially waterfowl (58, 59). Glomeromycota and Mucoromycota have proved to be important arbuscular mycorrhizal fungi (60). Genus *Basidiobolus* (Phylum Zoopagomycota) has a rich range of secondary metabolic genes involved in producing terpene cyclase, surfactin-like, and siderophore (61). The abundance of the fungal phyla mentioned above decreased significantly in the diarrheal group, revealing that diarrhea had a significant effect on the gut mycobiota.

At the genus levels, the composition and relative abundance of the gut fungal flora in the healthy and diarrheal groups also showed significant differences. The relative abundance of *Wickerhamomyces, Alternaria, Penicillium, Cystofilobasidium*, and *Filobasidium* in the diarrheal group was significantly higher than in the healthy group. *Wickerhamomyces* was reported to be significantly increased in ulcerative colitis patients and animals with colitis induced by dextran sulfate sodium (62, 63). Two genera were positively correlated to *Wickerhamomyces*, i.e., *Meyerozyma* and *Rhinocladiel*la. *Meyerozyma* was dramatically increased in type 2 short bowel syndrome rats (64). Remarkably, *Meyerozyma caribbica*, which was recognized as a gut fungi associated with diarrhea, has been confirmed to cause onychomycosis (65). Many fungi in the genus *Rhinocladiella* (e.g., *Rhinocladiella mackenziei, Rhinocladiella aquaspersa, Rhinocladiella simili*s) are pathogens of various diseases in humans and animals (66–68). A variety of secondary metabolites produced by small-spored *Alternaria* species have chronic or acute toxic effects of mutagenic, carcinogenicity, and teratogenicity in humans and animals (69). Fungi in the genus *Penicillium, such as Penicillium expansum, Penicillinum citrinum*, and *Penicillium islandicum*, could produce some harmful mycotoxins and carcinogenic compounds, such as verrucosidin, ochratoxin A, citrinine, patulin, citreoviridin, penicillic acid, and other toxic secondary metabolites, and then cause humans and animals various diseases (70–72). *Cystofilobasidiaceae* family was overrepresented in Crohn's disease (73). *Filobasidium magnum* and *Filobasidium uniguttulatum* were pathogens of otomycosis and bovine mastitis (74, 75). The appearance and changes in the above fungi in the gut of Baer's pochard were reported for the first time. LEfSe and Metastats analyses between healthy and diarrheal groups further identified several fungi closely associated with diarrhea. Abundances of *Wickerhamomyces, Meyerozyma*, and *Rhinocladiella* with positive correlations were significantly increased in the diarrheal group. LEfSe result showed that the relative abundance of *Wickerhamomyces anomalus* and *Meyerozyma caribbica* were significantly higher in the diarrheal group. It has been reported that *Wickerhamomyces anomalus* is a zoonotic pathogen that can cause severe iatrogenic infections and multiple clinical symptoms in animals (76, 77). *Meyerozyma caribbica* is a gut fungus associated with diarrhea (65). The significant increase of *Rhinocladiella similis* in the diarrheal group can cause chromoblastomycosis in humans (66, 78). Based on the analysis of the above results, it was speculated that these three fungi are the main pathogens causing diarrhea in the study subjects.

The microbial interaction (e.g., bacteria–fungi interaction) is important for keeping microbial homeostasis (79). Bacteria or bacterial metabolites can regulate the pathogenic properties and virulence of gut fungi. In contrast, fungi can protect themselves by secreting certain substances, forming biofilms, or building connections with other bacteria (80). Moreover, gut fungi are involved in the construction of the host gut microflora and the development of the immune system and then affect gut function and the physiological functions of other parenteral organs such as the liver, lungs, and brain (81). Fungal dysbiosis may increase allergies and inflammation, especially in the gut (82). Xi et al. (83) reported that the dominant genera in the diarrheal Baer's pochard were *Planococcus, Psychrobacter*, and *Exiguobacterium*. The abundances of beneficial bacteria such as *Alloprevotella, Veillonella, Parabacteroides, Prevotellaceae, uncultured_bacterium_f_Lachnospiraceae, Carnobacterium, Ruminococcaceae_UCG-002*, and *Ruminococcaceae_UCG-014* were significantly reduced in the gut of diarrheal Baer's pochard (83). *Meyerozyma guilliermondii* in the human gut can induce liver synthesis of more prostaglandin E_2_ to develop chronic alcoholic hepatic steatosis. Oral administration of water-insoluble polysaccharides could significantly increase the abundance of *Lachnospiraceae* and decrease the abundance of *Meyerozyma guilliermondii* (84). *Psychrobacter* and *Penicillium* were identified to coexist in a clinical microbiology laboratory of a university teaching hospital (85). Both *Penicillium* spp. (*Penicillium olsonii, Penicillium brevicompactum*, and *Penicillium cavernicola*), and *Psychrobacter* sp. (*Psychrobacter glacincola*) are capable of producing highly active protease and lipase (86), indicating that the two microorganisms may have synergistic action in the related function. To clarify how complex interactions between bacteria and fungi affect host health states, studies of the community composition of bacteria and fungi in the host gut, the association between bacterial and fungal taxa in the microbiome, and the relationship between bacterial–fungal disorders and host disease are required. Moreover, the effects of some limiting factors in this study, such as gender, age, and sample size, on the results also need to be considered in subsequent studies.

## Conclusion

In conclusion, this study analyzed the relationship between diarrhea and the diversity and composition of gut mycobiota in healthy and diarrheal Baer's pochard based on the high-throughput sequencing technique. Results showed significantly decreased gut fungal diversity of diarrheal Baer's pochard, and its composition also changed significantly. Moreover, the present study identified several fungi that may be associated with the occurrence and development of diarrhea in Baer's pochard, laying the theoretical basis for monitoring and formulating prevention and control measures for the infectious diseases of Baer's pochard.

## Data Availability Statement

The datasets presented in this study can be found in online repositories. The names of the repository/repositories and accession number(s) can be found below: https://www.ncbi.nlm.nih.gov/sra/PRJNA753894.

## Ethics Statement

The animal study was reviewed and approved by Ethics Committee of the Shangqiu Normal University.

## Author Contributions

LX conceived and designed the experiments and wrote the manuscript. YS, ZL, and JZ contributed to the sample collection and reagent preparation. XQ analyzed the data. JH revised the manuscript. All authors reviewed the manuscript.

## Funding

This study was supported by the Screening and Application of Anti-Foodborne Campylobacter Phage (Key Scientific Research Projects of Higher Education Institutions in Henan Province: 21B230008) and the study of PYY3-36 Active Immunization on Nutritional Physiology of Rats (Key Scientific Research Projects of Higher Education Institutions in Henan Province: 15A230028).

## Conflict of Interest

The authors declare that the research was conducted in the absence of any commercial or financial relationships that could be construed as a potential conflict of interest.

## Publisher's Note

All claims expressed in this article are solely those of the authors and do not necessarily represent those of their affiliated organizations, or those of the publisher, the editors and the reviewers. Any product that may be evaluated in this article, or claim that may be made by its manufacturer, is not guaranteed or endorsed by the publisher.
