# Supplementary material for: Gut Microbial Alterations in Diarrheal Baer's Pochards (Aythya baeri)
Source: Front Vet Sci. 2021 Oct 14;8:756486. doi: 10.3389/fvets.2021.756486 (PMC8551490; doi:10.3389/fvets.2021.756486)
Supplement: Supplementary file 1 [file Table_1.pdf]

Supplementary Table 1 Significant alterations in the gut fungal compositions at the genus levels.

Genus	Mean (C)	Variance (C)	Mean (D)	Variance (D)	*p* value
Achroiostachys	0.00E+00	0.00E+00	1.36E-04	1.49E-07	0.001
Armillaria	0.00E+00	0.00E+00	2.40E-04	1.96E-07	0.001
Bimuria	0.00E+00	0.00E+00	3.54E-04	9.90E-07	0.001
Clathrosphaerina	0.00E+00	0.00E+00	1.19E-04	1.14E-07	0.001
Clavaria	0.00E+00	0.00E+00	2.66E-04	5.64E-07	0.001
Claviceps	0.00E+00	0.00E+00	1.70E-04	1.20E-07	0.001
Clitopilus	0.00E+00	0.00E+00	1.89E-04	2.85E-07	0.001
Diaporthe	0.00E+00	0.00E+00	1.09E-04	6.42E-08	0.001
Kockovaella	0.00E+00	0.00E+00	1.20E-04	5.36E-08	0.001
Lachancea	0.00E+00	0.00E+00	8.84E-04	5.92E-06	0.001
Lachnum	0.00E+00	0.00E+00	4.48E-04	1.61E-06	0.001
Leptoxyphium	0.00E+00	0.00E+00	1.44E-04	1.63E-07	0.001
Membranomyces	0.00E+00	0.00E+00	1.71E-04	1.34E-07	0.001
Monodictys	0.00E+00	0.00E+00	1.14E-04	8.40E-08	0.001
Panaeolus	0.00E+00	0.00E+00	1.07E-04	9.19E-08	0.001
Phaeoannellomyces	0.00E+00	0.00E+00	4.01E-04	5.51E-07	0.001
Sarcinomyces	0.00E+00	0.00E+00	2.05E-04	3.36E-07	0.001
Scleroramularia	0.00E+00	0.00E+00	3.55E-04	1.01E-06	0.001
Stagonospora	0.00E+00	0.00E+00	1.73E-04	2.39E-07	0.001
Starmerella	0.00E+00	0.00E+00	3.21E-04	8.13E-07	0.001
Sympodiella	0.00E+00	0.00E+00	1.09E-04	9.55E-08	0.001
Taphrina	0.00E+00	0.00E+00	1.05E-04	7.93E-08	0.001
Trichaleurina	0.00E+00	0.00E+00	1.58E-04	1.99E-07	0.001
Tylopilus	0.00E+00	0.00E+00	1.10E-04	9.68E-08	0.001
Westerdykella	0.00E+00	0.00E+00	4.06E-04	1.32E-06	0.001
Wickerhamiella	0.00E+00	0.00E+00	5.00E-04	1.98E-06	0.001
Xylaria	0.00E+00	0.00E+00	1.35E-04	1.46E-07	0.001
Zopfiella	0.00E+00	0.00E+00	2.99E-04	7.07E-07	0.001
Rhinocladiella	2.18E-05	3.81E-09	5.71E-03	6.76E-05	0.001
Remersonia	2.04E-03	7.44E-07	5.34E-04	4.81E-07	0.003
Trichocladium	1.15E-03	1.09E-06	5.19E-06	2.15E-10	0.004
Fusicolla	2.67E-03	4.80E-06	5.54E-04	4.93E-07	0.005
Tetracladium	1.34E-03	7.78E-07	3.51E-04	1.59E-07	0.016
Torula	6.30E-04	1.95E-07	1.84E-04	4.32E-08	0.018
Condenascus	7.47E-03	2.01E-05	2.39E-03	7.61E-06	0.019
Neooccultibambusa	2.33E-04	1.15E-07	9.49E-06	7.20E-10	0.019
Solicoccozyma	1.66E-03	1.88E-06	4.08E-04	2.62E-07	0.019
Podosphaera	3.11E-04	1.45E-07	1.73E-06	2.39E-11	0.020
Pseudogymnoascus	1.82E-06	2.65E-11	1.32E-04	5.12E-08	0.020
Sebacina	1.43E-02	2.19E-04	2.74E-03	9.12E-06	0.020

Mortierella	2.81E-02	1.04E-04	1.32E-02	1.70E-04	0.023
Leohumicola	3.54E-03	1.90E-05	8.69E-04	1.23E-06	0.024
Microdochium	2.40E-03	6.05E-06	5.09E-04	1.88E-07	0.024
Humicola	6.62E-03	1.96E-05	2.14E-03	5.79E-06	0.026
Phaeosphaeria	4.03E-04	3.05E-07	2.41E-05	3.98E-09	0.027
Hygrocybe	1.70E-02	8.62E-04	2.30E-03	8.33E-06	0.028
Meyerozyma	1.34E-03	6.86E-06	2.87E-02	2.17E-03	0.030
Lepiota	2.88E-03	6.69E-06	5.76E-04	5.26E-07	0.031
Cephalotrichum	8.04E-04	9.63E-07	1.02E-04	3.68E-08	0.032
Boletus	9.52E-04	1.52E-06	5.17E-05	9.40E-09	0.038
Podospora	1.14E-03	7.07E-07	4.18E-04	1.96E-07	0.039
Lectera	3.47E-03	5.04E-06	1.29E-03	2.44E-06	0.041
Achroceratosphaeria	7.52E-03	6.32E-05	1.61E-03	3.93E-06	0.044
Echria	3.06E-03	9.55E-06	6.21E-04	1.35E-06	0.045
Bullera	8.21E-05	3.39E-08	8.98E-04	1.56E-06	0.048
Paraphaeosphaeria	5.01E-04	8.47E-07	2.04E-05	1.95E-09	0.049
Agrocybe	1.57E-04	1.98E-07	0.00E+00	0.00E+00	0.001
Ballistosporomyces	1.61E-04	2.07E-07	0.00E+00	0.00E+00	0.001
Brycekendrickomyces	1.27E-04	1.30E-07	0.00E+00	0.00E+00	0.001
Campylocarpon	1.92E-04	2.31E-07	0.00E+00	0.00E+00	0.001
Coniophora	1.04E-04	8.64E-08	0.00E+00	0.00E+00	0.001
Coprinus	1.36E-03	1.48E-05	0.00E+00	0.00E+00	0.001
Eleutheromyces	1.15E-04	1.05E-07	0.00E+00	0.00E+00	0.001
Geopora	2.58E-04	3.40E-07	0.00E+00	0.00E+00	0.001
Gliocephalotrichum	1.57E-04	1.98E-07	0.00E+00	0.00E+00	0.001
Lasiodiplodia	1.44E-04	1.62E-07	0.00E+00	0.00E+00	0.001
Leotia	3.01E-04	3.91E-07	0.00E+00	0.00E+00	0.001
Leptodiscella	2.44E-04	2.65E-07	0.00E+00	0.00E+00	0.001
Limonomyces	1.40E-04	1.38E-07	0.00E+00	0.00E+00	0.001
Madurella	1.03E-04	7.25E-08	0.00E+00	0.00E+00	0.001
Minimedusa	1.09E-04	9.58E-08	0.00E+00	0.00E+00	0.001
Minutisphaera	2.05E-04	3.37E-07	0.00E+00	0.00E+00	0.001
Neoascochyta	3.20E-04	4.21E-07	0.00E+00	0.00E+00	0.001
Neodactylaria	2.55E-04	5.22E-07	0.00E+00	0.00E+00	0.001
Nothodactylaria	1.29E-04	6.22E-08	0.00E+00	0.00E+00	0.001
Parasola	2.48E-04	3.91E-07	0.00E+00	0.00E+00	0.001
Penicillifer	1.54E-04	1.90E-07	0.00E+00	0.00E+00	0.001
Peniophorella	4.64E-04	1.41E-06	0.00E+00	0.00E+00	0.001
Phaeobotryon	1.47E-04	1.09E-07	0.00E+00	0.00E+00	0.001
Pilidium	1.48E-04	7.86E-08	0.00E+00	0.00E+00	0.001
Pisolithus	5.19E-04	8.06E-07	0.00E+00	0.00E+00	0.001
Powellomyces	1.15E-04	1.05E-07	0.00E+00	0.00E+00	0.001
Pseudaleuria	2.27E-04	3.90E-07	0.00E+00	0.00E+00	0.001
Rhizoctonia	1.83E-04	1.75E-07	0.00E+00	0.00E+00	0.001

Rotiferophthora	1.11E-04	9.88E-08	0.00E+00	0.00E+00	0.001
Sphaerellopsis	1.76E-04	2.42E-07	0.00E+00	0.00E+00	0.001
Sporisorium	1.25E-04	1.21E-07	0.00E+00	0.00E+00	0.001
Subsessila	2.03E-04	3.30E-07	0.00E+00	0.00E+00	0.001
Symbiotaphrina	4.52E-04	7.72E-07	0.00E+00	0.00E+00	0.001
Thermothielavioides	2.16E-04	3.73E-07	0.00E+00	0.00E+00	0.001
Towyspora	2.53E-04	2.37E-07	0.00E+00	0.00E+00	0.001
Tylospora	1.17E-04	1.10E-07	0.00E+00	0.00E+00	0.001
Virgaria	1.84E-04	2.72E-07	0.00E+00	0.00E+00	0.001
Conocybe	9.51E-04	6.80E-06	1.58E-06	2.00E-11	0.007
Acremonium	7.48E-03	7.57E-06	2.33E-03	5.00E-06	0.001

Supplementary Table 2 Edge property analysis among different fungal genera

**Source**	**Target**	**weight**	**color**
Wickerhamomyces	*Penicillium*	0.6706	negative
Wickerhamomyces	*Fusarium*	0.6088	negative
Wickerhamomyces	*Mortierella*	0.6941	negative
Wickerhamomyces	*Aspergillus*	0.7029	negative
Wickerhamomyces	*Sagenomella*	0.5912	negative
Wickerhamomyces	*Meyerozyma*	0.6941	positive
Wickerhamomyces	*Eleutherascus*	0.5382	negative
Wickerhamomyces	*Saitozyma*	0.55	negative
Wickerhamomyces	*Triangularia*	0.8794	negative
Wickerhamomyces	*Sebacina*	0.8794	negative
Wickerhamomyces	*Russula*	0.7588	negative
Wickerhamomyces	*Condenascus*	0.8147	negative
Wickerhamomyces	*Acremonium*	0.6	negative
Wickerhamomyces	*Achroceratosphaeria*	0.65	negative
Wickerhamomyces	*Humicola*	0.8294	negative
Wickerhamomyces	*Trichoderma*	0.6706	negative
Wickerhamomyces	*Arxiella*	0.7195	negative
Wickerhamomyces	*Gibellulopsis*	0.6206	negative
Wickerhamomyces	*Botryotrichum*	0.5618	negative
Wickerhamomyces	*Rhinocladiella*	0.5451	positive
Wickerhamomyces	*Candida*	0.6706	negative
Wickerhamomyces	*Lectera*	0.5618	negative
Wickerhamomyces	*Stolonocarpus*	0.6445	negative
Wickerhamomyces	*Leohumicola*	0.5294	negative
Wickerhamomyces	*Thermomyces*	0.5559	negative
Wickerhamomyces	*Lepiota*	0.7994	negative
Wickerhamomyces	*Fusicolla*	0.5615	negative
Wickerhamomyces	*Arthrographis*	0.5553	negative

Wickerhamomyces	*Ceratobasidium*	0.5422	negative
Wickerhamomyces	*Remersonia*	0.5147	negative
Wickerhamomyces	*Xerochrysium*	0.5319	negative
Wickerhamomyces	*Mycoarthris*	0.6657	negative
Wickerhamomyces	*Exophiala*	0.6637	negative
Wickerhamomyces	*Tomentella*	0.5408	negative
Alternaria	*Cystofilobasidium*	0.5059	positive
Alternaria	*Sagenomella*	0.5147	negative
Alternaria	*Chaetomium*	0.5176	negative
Alternaria	*Trichosporon*	0.5912	negative
Alternaria	*Sebacina*	0.5647	negative
Alternaria	*Russula*	0.5559	negative
Alternaria	*Schizothecium*	0.6343	negative
Alternaria	*Acremonium*	0.6647	negative
Alternaria	*Arthrinium*	0.6284	positive
Alternaria	*Achroceratosphaeria*	0.5559	negative
Alternaria	*Humicola*	0.5206	negative
Alternaria	*Metarhizium*	0.6294	negative
Alternaria	*Curvularia*	0.5636	positive
Alternaria	*Tricholoma*	0.5887	negative
Alternaria	*Kazachstania*	0.6461	negative
Alternaria	*Fusicolla*	0.5156	negative
Alternaria	*Iodophanus*	0.568	negative
Alternaria	*Tomentella*	0.5215	negative
Alternaria	*Colletotrichum*	0.5151	negative
Penicillium	*Talaromyces*	0.6471	positive
Penicillium	*Aspergillus*	0.6265	positive
Penicillium	*Meyerozyma*	0.5088	negative
Penicillium	*Triangularia*	0.5941	positive
Penicillium	*Sebacina*	0.6324	positive
Penicillium	*Russula*	0.6029	positive
Penicillium	*Condenascus*	0.6324	positive
Penicillium	*Acremonium*	0.5971	positive
Penicillium	*Achroceratosphaeria*	0.5206	positive
Penicillium	*Humicola*	0.6441	positive
Penicillium	*Trichoderma*	0.5588	positive
Penicillium	*Botryotrichum*	0.5235	positive
Penicillium	*Gymnoascus*	0.5378	positive
Penicillium	*Stolonocarpus*	0.7156	positive
Penicillium	*Myrmecridium*	0.6676	positive
Penicillium	*Thermomyces*	0.6294	positive
Penicillium	*Lepiota*	0.5221	positive
Penicillium	*Arthrographis*	0.6299	positive
Penicillium	*Tomentella*	0.5096	positive

Penicillium	*Colletotrichum*	0.4989	positive
Fusarium	*Filobasidium*	0.6353	negative
Fusarium	*Mortierella*	0.65	positive
Fusarium	*Aspergillus*	0.5029	positive
Fusarium	*Meyerozyma*	0.7088	negative
Fusarium	*Saitozyma*	0.5941	positive
Fusarium	*Hygrocybe*	0.6029	positive
Fusarium	*Triangularia*	0.5824	positive
Fusarium	*Sebacina*	0.6588	positive
Fusarium	*Russula*	0.6941	positive
Fusarium	*Condenascus*	0.6059	positive
Fusarium	*Acremonium*	0.7176	positive
Fusarium	*Humicola*	0.5735	positive
Fusarium	*Nigrospora*	0.5029	positive
Fusarium	*Trichoderma*	0.5059	positive
Fusarium	*Rhinocladiella*	0.622	negative
Fusarium	*Metarhizium*	0.5559	positive
Fusarium	*Candida*	0.6853	positive
Fusarium	*Lectera*	0.85	positive
Fusarium	*Leohumicola*	0.8824	positive
Fusarium	*Myrmecridium*	0.5824	positive
Fusarium	*Clonostachys*	0.7088	positive
Fusarium	*Lepiota*	0.5634	positive
Fusarium	*Microdochium*	0.6706	positive
Fusarium	*Xerochrysium*	0.523	positive
Fusarium	*Exophiala*	0.5782	positive
Fusarium	*Tomentella*	0.5793	positive
Cystofilobasidium	*Mortierella*	0.5941	negative
Cystofilobasidium	*Sagenomella*	0.5265	negative
Cystofilobasidium	*Eleutherascus*	0.6	negative
Cystofilobasidium	*Saitozyma*	0.6265	negative
Cystofilobasidium	*Chaetomium*	0.6235	negative
Cystofilobasidium	*Trichosporon*	0.7	negative
Cystofilobasidium	*Triangularia*	0.6941	negative
Cystofilobasidium	*Sebacina*	0.7029	negative
Cystofilobasidium	*Russula*	0.6618	negative
Cystofilobasidium	*Schizothecium*	0.7035	negative
Cystofilobasidium	*Archaeorhizomyces*	0.7118	negative
Cystofilobasidium	*Condenascus*	0.5971	negative
Cystofilobasidium	*Acremonium*	0.6206	negative
Cystofilobasidium	*Arthrinium*	0.6431	positive
Cystofilobasidium	*Achroceratosphaeria*	0.5882	negative
Cystofilobasidium	*Humicola*	0.6676	negative
Cystofilobasidium	*Trichoderma*	0.75	negative

Cystofilobasidium	*Leptobacillium*	0.6529	negative
Cystofilobasidium	*Arxiella*	0.5523	negative
Cystofilobasidium	*Gibellulopsis*	0.7765	negative
Cystofilobasidium	*Botryotrichum*	0.65	negative
Cystofilobasidium	*Metarhizium*	0.5676	negative
Cystofilobasidium	*Oidiodendron*	0.6313	negative
Cystofilobasidium	*Stolonocarpus*	0.5037	negative
Cystofilobasidium	*Cercospora*	0.5754	negative
Cystofilobasidium	*Mycothermus*	0.5605	negative
Cystofilobasidium	*Fusicolla*	0.6963	negative
Cystofilobasidium	*Ceratobasidium*	0.6563	negative
Cystofilobasidium	*Remersonia*	0.6765	negative
Cystofilobasidium	*Xerochrysium*	0.5008	negative
Cystofilobasidium	*Mycoarthris*	0.5284	negative
Cystofilobasidium	*Colletotrichum*	0.6093	negative
Cladosporium	*Filobasidium*	0.6294	positive
Cladosporium	*Thelebolus*	0.5843	negative
Cladosporium	*Thermomyces*	0.5794	negative
Cladosporium	*Tricholoma*	0.4974	negative
Cladosporium	*Tomentella*	0.5052	negative
Filobasidium	*Mortierella*	0.6471	negative
Filobasidium	*Meyerozyma*	0.5647	positive
Filobasidium	*Eleutherascus*	0.5206	negative
Filobasidium	*Saitozyma*	0.6147	negative
Filobasidium	*Acremonium*	0.5059	negative
Filobasidium	*Rhinocladiella*	0.6377	positive
Filobasidium	*Candida*	0.5676	negative
Filobasidium	*Lectera*	0.75	negative
Filobasidium	*Leohumicola*	0.6147	negative
Filobasidium	*Echria*	0.6471	negative
Filobasidium	*Microdochium*	0.55	negative
Filobasidium	*Paraphoma*	0.5471	negative
Talaromyces	*Aspergillus*	0.5324	positive
Talaromyces	*Sagenomella*	0.5647	positive
Talaromyces	*Acremonium*	0.5088	positive
Talaromyces	*Candida*	0.5206	positive
Talaromyces	*Curvularia*	0.6152	negative
Talaromyces	*Myrmecridium*	0.5412	positive
Talaromyces	*Thermomyces*	0.5559	positive
Talaromyces	*Lepiota*	0.5103	positive
Talaromyces	*Tricholoma*	0.5136	positive
Talaromyces	*Kazachstania*	0.6431	positive
Talaromyces	*Arthrographis*	0.818	positive
Talaromyces	*Tomentella*	0.5585	positive

Mortierella	*Aspergillus*	0.7088	positive
Mortierella	*Meyerozyma*	0.6324	negative
Mortierella	*Eleutherascus*	0.5441	positive
Mortierella	*Saitozyma*	0.6059	positive
Mortierella	*Hygrocybe*	0.5794	positive
Mortierella	*Triangularia*	0.6706	positive
Mortierella	*Sebacina*	0.7059	positive
Mortierella	*Russula*	0.6147	positive
Mortierella	*Archaeorhizomyces*	0.6382	positive
Mortierella	*Condenascus*	0.8059	positive
Mortierella	*Acremonium*	0.7324	positive
Mortierella	*Achroceratosphaeria*	0.6265	positive
Mortierella	*Humicola*	0.7912	positive
Mortierella	*Trichoderma*	0.6118	positive
Mortierella	*Leptobacillium*	0.7235	positive
Mortierella	*Arxiella*	0.7225	positive
Mortierella	*Gibellulopsis*	0.5265	positive
Mortierella	*Rhodotorula*	0.5401	negative
Mortierella	*Botryotrichum*	0.5029	positive
Mortierella	*Pyrenochaetopsis*	0.5559	positive
Mortierella	*Rhinocladiella*	0.7069	negative
Mortierella	*Metarhizium*	0.6765	positive
Mortierella	*Candida*	0.7647	positive
Mortierella	*Lectera*	0.6676	positive
Mortierella	*Stolonocarpus*	0.6771	positive
Mortierella	*Leohumicola*	0.7	positive
Mortierella	*Myrmecridium*	0.5324	positive
Mortierella	*Wallemia*	0.5	positive
Mortierella	*Clonostachys*	0.5	positive
Mortierella	*Lepiota*	0.6224	positive
Mortierella	*Fusicolla*	0.8104	positive
Mortierella	*Microdochium*	0.5118	positive
Mortierella	*Paraphoma*	0.8029	positive
Mortierella	*Remersonia*	0.6147	positive
Mortierella	*Exophiala*	0.6136	positive
Mortierella	*Colletotrichum*	0.6152	positive
Aspergillus	*Sagenomella*	0.5088	positive
Aspergillus	*Meyerozyma*	0.5029	negative
Aspergillus	*Saitozyma*	0.5206	positive
Aspergillus	*Triangularia*	0.6824	positive
Aspergillus	*Sebacina*	0.6941	positive
Aspergillus	*Russula*	0.6765	positive
Aspergillus	*Condenascus*	0.7206	positive
Aspergillus	*Acremonium*	0.5882	positive

Aspergillus	*Achroceratosphaeria*	0.7	positive
Aspergillus	*Humicola*	0.7765	positive
Aspergillus	*Trichoderma*	0.5647	positive
Aspergillus	*Leptobacillium*	0.6618	positive
Aspergillus	*Arxiella*	0.827	positive
Aspergillus	*Cystobasidium*	0.5695	negative
Aspergillus	*Botryotrichum*	0.5176	positive
Aspergillus	*Metarhizium*	0.6118	positive
Aspergillus	*Candida*	0.7824	positive
Aspergillus	*Lectera*	0.5176	positive
Aspergillus	*Gymnoascus*	0.5867	positive
Aspergillus	*Stolonocarpus*	0.6371	positive
Aspergillus	*Myrmecridium*	0.6324	positive
Aspergillus	*Thermomyces*	0.5912	positive
Aspergillus	*Wallemia*	0.6824	positive
Aspergillus	*Lepiota*	0.764	positive
Aspergillus	*Kazachstania*	0.5195	positive
Aspergillus	*Thermoascus*	0.6402	positive
Aspergillus	*Arthrographis*	0.7762	positive
Aspergillus	*Paraphoma*	0.7294	positive
Aspergillus	*Xerochrysium*	0.6578	positive
Aspergillus	*Exophiala*	0.6932	positive
Aspergillus	*Tomentella*	0.6178	positive
Aspergillus	*Colletotrichum*	0.6887	positive
Sagenomella	*Saitozyma*	0.6824	positive
Sagenomella	*Triangularia*	0.7559	positive
Sagenomella	*Sebacina*	0.6559	positive
Sagenomella	*Russula*	0.6971	positive
Sagenomella	*Condenascus*	0.5088	positive
Sagenomella	*Achroceratosphaeria*	0.5265	positive
Sagenomella	*Humicola*	0.5559	positive
Sagenomella	*Trichoderma*	0.5853	positive
Sagenomella	*Leptobacillium*	0.5	positive
Sagenomella	*Arxiella*	0.5613	positive
Sagenomella	*Gibellulopsis*	0.7	positive
Sagenomella	*Botryotrichum*	0.5147	positive
Sagenomella	*Candida*	0.5971	positive
Sagenomella	*Cercospora*	0.7241	positive
Sagenomella	*Mycothermus*	0.7817	positive
Sagenomella	*Lepiota*	0.6106	positive
Sagenomella	*Tricholoma*	0.7005	positive
Sagenomella	*Kazachstania*	0.7638	positive
Sagenomella	*Stachybotrys*	0.5693	positive
Sagenomella	*Arthrographis*	0.6329	positive

Sagenomella	*Ceratobasidium*	0.5274	positive
Sagenomella	*Xerochrysium*	0.5156	positive
Sagenomella	*Mycoarthris*	0.5732	positive
Sagenomella	*Exophiala*	0.5959	positive
Meyerozyma	*Eleutherascus*	0.6265	negative
Meyerozyma	*Triangularia*	0.6353	negative
Meyerozyma	*Sebacina*	0.7794	negative
Meyerozyma	*Russula*	0.6471	negative
Meyerozyma	*Schizothecium*	0.5004	negative
Meyerozyma	*Condenascus*	0.5647	negative
Meyerozyma	*Acremonium*	0.7794	negative
Meyerozyma	*Achroceratosphaeria*	0.5265	negative
Meyerozyma	*Humicola*	0.7118	negative
Meyerozyma	*Rhinocladiella*	0.6833	positive
Meyerozyma	*Metarhizium*	0.5235	negative
Meyerozyma	*Candida*	0.5735	negative
Meyerozyma	*Lectera*	0.6265	negative
Meyerozyma	*Stolonocarpus*	0.7052	negative
Meyerozyma	*Leohumicola*	0.7294	negative
Meyerozyma	*Myrmecridium*	0.5853	negative
Meyerozyma	*Thermomyces*	0.5118	negative
Meyerozyma	*Fusicolla*	0.6311	negative
Meyerozyma	*Microdochium*	0.6176	negative
Meyerozyma	*Arthrographis*	0.5314	negative
Meyerozyma	*Remersonia*	0.5824	negative
Meyerozyma	*Xerochrysium*	0.5911	negative
Meyerozyma	*Tomentella*	0.603	negative
Meyerozyma	*Colletotrichum*	0.6269	negative
Eleutherascus	*Sebacina*	0.5206	positive
Eleutherascus	*Gibellulopsis*	0.6824	positive
Eleutherascus	*Botryotrichum*	0.5088	positive
Eleutherascus	*Rhinocladiella*	0.7226	negative
Eleutherascus	*Fusicolla*	0.5304	positive
Saitozyma	*Chaetomium*	0.6824	positive
Saitozyma	*Cladorrhinum*	0.5912	positive
Saitozyma	*Triangularia*	0.5971	positive
Saitozyma	*Sebacina*	0.6	positive
Saitozyma	*Russula*	0.55	positive
Saitozyma	*Archaeorhizomyces*	0.6441	positive
Saitozyma	*Condenascus*	0.5294	positive
Saitozyma	*Humicola*	0.5382	positive
Saitozyma	*Gibellulopsis*	0.7294	positive
Saitozyma	*Metarhizium*	0.5	positive
Saitozyma	*Candida*	0.7382	positive

Saitozyma	*Lectera*	0.6971	positive
Saitozyma	*Oidiodendron*	0.6696	positive
Saitozyma	*Leohumicola*	0.6529	positive
Saitozyma	*Cercospora*	0.5636	positive
Saitozyma	*Mycothermus*	0.531	positive
Saitozyma	*Tricholoma*	0.7108	positive
Saitozyma	*Microdochium*	0.6	positive
Saitozyma	*Xerochrysium*	0.4978	positive
Saitozyma	*Exophiala*	0.7021	positive
Saitozyma	*Tomentella*	0.5111	positive
Chaetomium	*Trichosporon*	0.6588	positive
Chaetomium	*Archaeorhizomyces*	0.7794	positive
Chaetomium	*Leptobacillium*	0.5412	positive
Chaetomium	*Metarhizium*	0.5206	positive
Chaetomium	*Oidiodendron*	0.5254	positive
Chaetomium	*Tricholoma*	0.5857	positive
Chaetomium	*Paraphoma*	0.5088	positive
Chaetomium	*Exophiala*	0.5546	positive
Chaetomium	*Iodophanus*	0.5738	positive
Chaetomium	*Submersisphaeria*	0.5315	positive
Trichosporon	*Sebacina*	0.5059	positive
Trichosporon	*Schizothecium*	0.752	positive
Trichosporon	*Archaeorhizomyces*	0.6941	positive
Trichosporon	*Arthrinium*	0.5651	negative
Trichosporon	*Achroceratosphaeria*	0.5971	positive
Trichosporon	*Humicola*	0.5147	positive
Trichosporon	*Trichoderma*	0.5941	positive
Trichosporon	*Leptobacillium*	0.5882	positive
Trichosporon	*Oidiodendron*	0.7285	positive
Trichosporon	*Cercospora*	0.5533	positive
Trichosporon	*Mycothermus*	0.649	positive
Trichosporon	*Stachybotrys*	0.7847	positive
Trichosporon	*Ceratobasidium*	0.52	positive
Trichosporon	*Mycoarthris*	0.621	positive
Trichosporon	*Exophiala*	0.5015	positive
Trichosporon	*Iodophanus*	0.5352	positive
Trichosporon	*Submersisphaeria*	0.5769	positive
Plectosphaerella	*Stachybotrys*	0.5693	positive
Cladorrhinum	*Papiliotrema*	0.7353	negative
Cladorrhinum	*Schizothecium*	0.5018	positive
Cladorrhinum	*Gibellulopsis*	0.5353	positive
Cladorrhinum	*Oidiodendron*	0.6431	positive
Cladorrhinum	*Microdochium*	0.5676	positive
Hygrocybe	*Acremonium*	0.7676	positive

Hygrocybe	*Leptobacillium*	0.5441	positive
Hygrocybe	*Metarhizium*	0.7647	positive
Hygrocybe	*Candida*	0.6971	positive
Hygrocybe	*Lectera*	0.8029	positive
Hygrocybe	*Leohumicola*	0.7853	positive
Hygrocybe	*Echria*	0.85	positive
Hygrocybe	*Clonostachys*	0.55	positive
Hygrocybe	*Tricholoma*	0.5681	positive
Hygrocybe	*Fusicolla*	0.5348	positive
Hygrocybe	*Microdochium*	0.9088	positive
Hygrocybe	*Paraphoma*	0.5588	positive
Hygrocybe	*Remersonia*	0.6882	positive
Triangularia	*Sebacina*	0.9294	positive
Triangularia	*Russula*	0.9235	positive
Triangularia	*Schizothecium*	0.5769	positive
Triangularia	*Archaeorhizomyces*	0.5529	positive
Triangularia	*Condenascus*	0.8794	positive
Triangularia	*Acremonium*	0.65	positive
Triangularia	*Arthrinium*	0.5784	negative
Triangularia	*Achroceratosphaeria*	0.7853	positive
Triangularia	*Humicola*	0.8912	positive
Triangularia	*Trichoderma*	0.8882	positive
Triangularia	*Leptobacillium*	0.5441	positive
Triangularia	*Arxiella*	0.818	positive
Triangularia	*Gibellulopsis*	0.7471	positive
Triangularia	*Botryotrichum*	0.8147	positive
Triangularia	*Candida*	0.6059	positive
Triangularia	*Oidiodendron*	0.4974	positive
Triangularia	*Gymnoascus*	0.6637	positive
Triangularia	*Stolonocarpus*	0.7734	positive
Triangularia	*Leohumicola*	0.5676	positive
Triangularia	*Cercospora*	0.5857	positive
Triangularia	*Myrmecridium*	0.6294	positive
Triangularia	*Mycothermus*	0.6755	positive
Triangularia	*Clonostachys*	0.5824	positive
Triangularia	*Lepiota*	0.8171	positive
Triangularia	*Kazachstania*	0.5828	positive
Triangularia	*Fusicolla*	0.6119	positive
Triangularia	*Stachybotrys*	0.5811	positive
Triangularia	*Thermoascus*	0.5063	positive
Triangularia	*Arthrographis*	0.5971	positive
Triangularia	*Ceratobasidium*	0.7763	positive
Triangularia	*Remersonia*	0.6294	positive
Triangularia	*Xerochrysium*	0.6385	positive

Triangularia	*Mycoarthris*	0.7046	positive
Triangularia	*Exophiala*	0.6637	positive
Triangularia	*Tomentella*	0.6296	positive
Triangularia	*Colletotrichum*	0.677	positive
Sebacina	*Russula*	0.9	positive
Sebacina	*Schizothecium*	0.7035	positive
Sebacina	*Archaeorhizomyces*	0.5412	positive
Sebacina	*Condenascus*	0.8382	positive
Sebacina	*Acremonium*	0.7735	positive
Sebacina	*Arthrinium*	0.6534	negative
Sebacina	*Achroceratosphaeria*	0.8382	positive
Sebacina	*Humicola*	0.9441	positive
Sebacina	*Trichoderma*	0.8294	positive
Sebacina	*Leptobacillium*	0.5412	positive
Sebacina	*Arxiella*	0.7404	positive
Sebacina	*Gibellulopsis*	0.6412	positive
Sebacina	*Botryotrichum*	0.7088	positive
Sebacina	*Rhinocladiella*	0.5388	negative
Sebacina	*Metarhizium*	0.5912	positive
Sebacina	*Candida*	0.6265	positive
Sebacina	*Lectera*	0.5294	positive
Sebacina	*Oidiodendron*	0.5578	positive
Sebacina	*Gymnoascus*	0.5615	positive
Sebacina	*Stolonocarpus*	0.7748	positive
Sebacina	*Leohumicola*	0.5824	positive
Sebacina	*Myrmecridium*	0.6706	positive
Sebacina	*Thermomyces*	0.5735	positive
Sebacina	*Mycothermus*	0.5929	positive
Sebacina	*Clonostachys*	0.55	positive
Sebacina	*Lepiota*	0.7847	positive
Sebacina	*Kazachstania*	0.6004	positive
Sebacina	*Fusicolla*	0.7052	positive
Sebacina	*Stachybotrys*	0.6077	positive
Sebacina	*Thermoascus*	0.5136	positive
Sebacina	*Arthrographis*	0.615	positive
Sebacina	*Ceratobasidium*	0.7156	positive
Sebacina	*Remersonia*	0.6353	positive
Sebacina	*Xerochrysium*	0.7334	positive
Sebacina	*Mycoarthris*	0.7493	positive
Sebacina	*Exophiala*	0.6873	positive
Sebacina	*Tomentella*	0.7363	positive
Sebacina	*Colletotrichum*	0.7314	positive
Papiliotrema	*Gibellulopsis*	0.5794	negative
Papiliotrema	*Oidiodendron*	0.5798	negative

Papiliotrema	*Remersonia*	0.5853	negative
Russula	*Schizothecium*	0.596	positive
Russula	*Archaeorhizomyces*	0.5353	positive
Russula	*Condenascus*	0.8029	positive
Russula	*Acremonium*	0.7324	positive
Russula	*Arthrinium*	0.5107	negative
Russula	*Achroceratosphaeria*	0.8088	positive
Russula	*Humicola*	0.8588	positive
Russula	*Trichoderma*	0.9235	positive
Russula	*Leptobacillium*	0.5588	positive
Russula	*Arxiella*	0.7284	positive
Russula	*Gibellulopsis*	0.5647	positive
Russula	*Botryotrichum*	0.8265	positive
Russula	*Metarhizium*	0.5324	positive
Russula	*Candida*	0.5941	positive
Russula	*Lectera*	0.5176	positive
Russula	*Gymnoascus*	0.7585	positive
Russula	*Stolonocarpus*	0.7867	positive
Russula	*Leohumicola*	0.6382	positive
Russula	*Cercospora*	0.6122	positive
Russula	*Myrmecridium*	0.8235	positive
Russula	*Mycothermus*	0.6755	positive
Russula	*Clonostachys*	0.7147	positive
Russula	*Lepiota*	0.7817	positive
Russula	*Tricholoma*	0.5048	positive
Russula	*Kazachstania*	0.6726	positive
Russula	*Fusicolla*	0.5082	positive
Russula	*Stachybotrys*	0.6342	positive
Russula	*Thermoascus*	0.7005	positive
Russula	*Arthrographis*	0.6896	positive
Russula	*Ceratobasidium*	0.7348	positive
Russula	*Remersonia*	0.5824	positive
Russula	*Xerochrysium*	0.6474	positive
Russula	*Mycoarthris*	0.6478	positive
Russula	*Exophiala*	0.7109	positive
Russula	*Tomentella*	0.7585	positive
Russula	*Colletotrichum*	0.7726	positive
Schizothecium	*Archaeorhizomyces*	0.5239	positive
Schizothecium	*Acremonium*	0.5254	positive
Schizothecium	*Arthrinium*	0.6436	negative
Schizothecium	*Achroceratosphaeria*	0.6372	positive
Schizothecium	*Humicola*	0.6313	positive
Schizothecium	*Trichoderma*	0.5931	positive
Schizothecium	*Gibellulopsis*	0.5342	positive

Schizothecium	*Oidiodendron*	0.827	positive
Schizothecium	*Mycothermus*	0.7114	positive
Schizothecium	*Fusicolla*	0.5797	positive
Schizothecium	*Stachybotrys*	0.6701	positive
Schizothecium	*Ceratobasidium*	0.6464	positive
Schizothecium	*Mycoarthris*	0.7424	positive
Schizothecium	*Tomentella*	0.5604	positive
Schizothecium	*Colletotrichum*	0.5427	positive
Archaeorhizomyces	*Condenascus*	0.6618	positive
Archaeorhizomyces	*Acremonium*	0.5029	positive
Archaeorhizomyces	*Arthrinium*	0.5578	negative
Archaeorhizomyces	*Achroceratosphaeria*	0.5765	positive
Archaeorhizomyces	*Humicola*	0.6529	positive
Archaeorhizomyces	*Trichoderma*	0.7265	positive
Archaeorhizomyces	*Leptobacillium*	0.6941	positive
Archaeorhizomyces	*Arxiella*	0.5284	positive
Archaeorhizomyces	*Gibellulopsis*	0.5647	positive
Archaeorhizomyces	*Metarhizium*	0.6118	positive
Archaeorhizomyces	*Oidiodendron*	0.6623	positive
Archaeorhizomyces	*Cercospora*	0.5151	positive
Archaeorhizomyces	*Fusicolla*	0.5896	positive
Archaeorhizomyces	*Stachybotrys*	0.5634	positive
Archaeorhizomyces	*Paraphoma*	0.5676	positive
Archaeorhizomyces	*Ceratobasidium*	0.4978	positive
Archaeorhizomyces	*Remersonia*	0.6088	positive
Archaeorhizomyces	*Exophiala*	0.7021	positive
Archaeorhizomyces	*Iodophanus*	0.5757	positive
Archaeorhizomyces	*Submersisphaeria*	0.6061	positive
Archaeorhizomyces	*Tomentella*	0.603	positive
Condenascus	*Acremonium*	0.7235	positive
Condenascus	*Arthrinium*	0.6284	negative
Condenascus	*Achroceratosphaeria*	0.7559	positive
Condenascus	*Humicola*	0.9	positive
Condenascus	*Trichoderma*	0.8324	positive
Condenascus	*Leptobacillium*	0.5676	positive
Condenascus	*Arxiella*	0.7911	positive
Condenascus	*Gibellulopsis*	0.5735	positive
Condenascus	*Rhodotorula*	0.5269	negative
Condenascus	*Botryotrichum*	0.7206	positive
Condenascus	*Rhinocladiella*	0.5796	negative
Condenascus	*Metarhizium*	0.6235	positive
Condenascus	*Candida*	0.6353	positive
Condenascus	*Lectera*	0.5529	positive
Condenascus	*Gymnoascus*	0.6845	positive

Condenascus	*Stolonocarpus*	0.7704	positive
Condenascus	*Leohumicola*	0.6118	positive
Condenascus	*Myrmecridium*	0.5794	positive
Condenascus	*Clonostachys*	0.5941	positive
Condenascus	*Lepiota*	0.7729	positive
Condenascus	*Kazachstania*	0.5107	positive
Condenascus	*Fusicolla*	0.6889	positive
Condenascus	*Thermoascus*	0.5843	positive
Condenascus	*Paraphoma*	0.5559	positive
Condenascus	*Ceratobasidium*	0.5689	positive
Condenascus	*Remersonia*	0.7235	positive
Condenascus	*Xerochrysium*	0.5659	positive
Condenascus	*Exophiala*	0.6991	positive
Condenascus	*Tomentella*	0.6622	positive
Condenascus	*Colletotrichum*	0.6829	positive
Acremonium	*Arthrinium*	0.596	negative
Acremonium	*Achroceratosphaeria*	0.6794	positive
Acremonium	*Humicola*	0.7735	positive
Acremonium	*Trichoderma*	0.65	positive
Acremonium	*Leptobacillium*	0.6559	positive
Acremonium	*Arxiella*	0.5314	positive
Acremonium	*Rhinocladiella*	0.5435	negative
Acremonium	*Metarhizium*	0.8971	positive
Acremonium	*Candida*	0.7059	positive
Acremonium	*Lectera*	0.7353	positive
Acremonium	*Gymnoascus*	0.523	positive
Acremonium	*Stolonocarpus*	0.7171	positive
Acremonium	*Leohumicola*	0.7676	positive
Acremonium	*Myrmecridium*	0.7	positive
Acremonium	*Echria*	0.5382	positive
Acremonium	*Clonostachys*	0.6735	positive
Acremonium	*Lepiota*	0.6018	positive
Acremonium	*Tricholoma*	0.5754	positive
Acremonium	*Kazachstania*	0.5725	positive
Acremonium	*Fusicolla*	0.763	positive
Acremonium	*Thermoascus*	0.5592	positive
Acremonium	*Microdochium*	0.6941	positive
Acremonium	*Arthrographis*	0.5911	positive
Acremonium	*Paraphoma*	0.5294	positive
Acremonium	*Remersonia*	0.8029	positive
Acremonium	*Xerochrysium*	0.52	positive
Acremonium	*Exophiala*	0.5634	positive
Acremonium	*Tomentella*	0.7348	positive
Acremonium	*Colletotrichum*	0.7167	positive

Arthrinium	*Achroceratosphaeria*	0.7226	negative
Arthrinium	*Humicola*	0.6858	negative
Arthrinium	*Trichoderma*	0.6431	negative
Arthrinium	*Arxiella*	0.5258	negative
Arthrinium	*Cystobasidium*	0.5133	positive
Arthrinium	*Metarhizium*	0.5018	negative
Arthrinium	*Stolonocarpus*	0.6019	negative
Arthrinium	*Lepiota*	0.4974	negative
Arthrinium	*Fusicolla*	0.811	negative
Arthrinium	*Stachybotrys*	0.5137	negative
Arthrinium	*Ceratobasidium*	0.6612	negative
Arthrinium	*Remersonia*	0.5592	negative
Arthrinium	*Mycoarthris*	0.5049	negative
Arthrinium	*Iodophanus*	0.6805	negative
Arthrinium	*Colletotrichum*	0.5714	negative
Achroceratosphaeria	*Humicola*	0.9176	positive
Achroceratosphaeria	*Trichoderma*	0.8088	positive
Achroceratosphaeria	*Leptobacillium*	0.6382	positive
Achroceratosphaeria	*Arxiella*	0.824	positive
Achroceratosphaeria	*Cystobasidium*	0.7167	negative
Achroceratosphaeria	*Rhodotorula*	0.5269	negative
Achroceratosphaeria	*Botryotrichum*	0.6147	positive
Achroceratosphaeria	*Metarhizium*	0.6029	positive
Achroceratosphaeria	*Candida*	0.5676	positive
Achroceratosphaeria	*Curvularia*	0.5548	negative
Achroceratosphaeria	*Oidiodendron*	0.5033	positive
Achroceratosphaeria	*Gymnoascus*	0.7689	positive
Achroceratosphaeria	*Stolonocarpus*	0.7689	positive
Achroceratosphaeria	*Myrmecridium*	0.7118	positive
Achroceratosphaeria	*Thermomyces*	0.5882	positive
Achroceratosphaeria	*Mycothermus*	0.5988	positive
Achroceratosphaeria	*Clonostachys*	0.5647	positive
Achroceratosphaeria	*Lepiota*	0.8614	positive
Achroceratosphaeria	*Kazachstania*	0.6755	positive
Achroceratosphaeria	*Fusicolla*	0.6163	positive
Achroceratosphaeria	*Stachybotrys*	0.7168	positive
Achroceratosphaeria	*Thermoascus*	0.6313	positive
Achroceratosphaeria	*Arthrographis*	0.6986	positive
Achroceratosphaeria	*Ceratobasidium*	0.6548	positive
Achroceratosphaeria	*Remersonia*	0.5765	positive
Achroceratosphaeria	*Xerochrysium*	0.7556	positive
Achroceratosphaeria	*Mycoarthris*	0.6926	positive
Achroceratosphaeria	*Exophiala*	0.7257	positive
Achroceratosphaeria	*Tomentella*	0.7985	positive

Achroceratosphaeria	*Colletotrichum*	0.8477	positive
Hannaella	*Gibellulopsis*	0.5235	positive
Humicola	*Trichoderma*	0.8559	positive
Humicola	*Leptobacillium*	0.6471	positive
Humicola	*Arxiella*	0.8329	positive
Humicola	*Cystobasidium*	0.6402	negative
Humicola	*Gibellulopsis*	0.5412	positive
Humicola	*Botryotrichum*	0.7147	positive
Humicola	*Rhinocladiella*	0.5686	negative
Humicola	*Metarhizium*	0.6588	positive
Humicola	*Candida*	0.6559	positive
Humicola	*Lectera*	0.5	positive
Humicola	*Oidiodendron*	0.5283	positive
Humicola	*Gymnoascus*	0.6948	positive
Humicola	*Stolonocarpus*	0.8326	positive
Humicola	*Leohumicola*	0.5294	positive
Humicola	*Myrmecridium*	0.7	positive
Humicola	*Thermomyces*	0.5971	positive
Humicola	*Mycothermus*	0.5546	positive
Humicola	*Clonostachys*	0.5794	positive
Humicola	*Lepiota*	0.8171	positive
Humicola	*Kazachstania*	0.6137	positive
Humicola	*Fusicolla*	0.7215	positive
Humicola	*Stachybotrys*	0.5929	positive
Humicola	*Thermoascus*	0.6269	positive
Humicola	*Arthrographis*	0.6478	positive
Humicola	*Paraphoma*	0.5324	positive
Humicola	*Ceratobasidium*	0.6519	positive
Humicola	*Remersonia*	0.6971	positive
Humicola	*Xerochrysium*	0.7348	positive
Humicola	*Mycoarthris*	0.6717	positive
Humicola	*Exophiala*	0.7463	positive
Humicola	*Tomentella*	0.803	positive
Humicola	*Colletotrichum*	0.8315	positive
Nigrospora	*Leohumicola*	0.5324	positive
Nigrospora	*Microdochium*	0.5529	positive
Trichoderma	*Leptobacillium*	0.5824	positive
Trichoderma	*Arxiella*	0.7165	positive
Trichoderma	*Gibellulopsis*	0.6088	positive
Trichoderma	*Botryotrichum*	0.8706	positive
Trichoderma	*Metarhizium*	0.5059	positive
Trichoderma	*Oidiodendron*	0.5224	positive
Trichoderma	*Gymnoascus*	0.7763	positive
Trichoderma	*Stolonocarpus*	0.7526	positive

Trichoderma	*Cercospora*	0.5887	positive
Trichoderma	*Myrmecridium*	0.7382	positive
Trichoderma	*Mycothermus*	0.649	positive
Trichoderma	*Clonostachys*	0.6765	positive
Trichoderma	*Lepiota*	0.6785	positive
Trichoderma	*Kazachstania*	0.5666	positive
Trichoderma	*Fusicolla*	0.5496	positive
Trichoderma	*Stachybotrys*	0.6844	positive
Trichoderma	*Thermoascus*	0.6755	positive
Trichoderma	*Arthrographis*	0.5254	positive
Trichoderma	*Ceratobasidium*	0.8163	positive
Trichoderma	*Remersonia*	0.6588	positive
Trichoderma	*Xerochrysium*	0.563	positive
Trichoderma	*Mycoarthris*	0.621	positive
Trichoderma	*Exophiala*	0.6637	positive
Trichoderma	*Iodophanus*	0.5216	positive
Trichoderma	*Tomentella*	0.7393	positive
Trichoderma	*Colletotrichum*	0.7535	positive
Leptobacillium	*Arxiella*	0.7314	positive
Leptobacillium	*Cystobasidium*	0.5107	negative
Leptobacillium	*Metarhizium*	0.7	positive
Leptobacillium	*Candida*	0.6912	positive
Leptobacillium	*Curvularia*	0.5666	negative
Leptobacillium	*Gymnoascus*	0.5289	positive
Leptobacillium	*Stolonocarpus*	0.5837	positive
Leptobacillium	*Cercospora*	0.5872	positive
Leptobacillium	*Myrmecridium*	0.6176	positive
Leptobacillium	*Wallemia*	0.5176	positive
Leptobacillium	*Lepiota*	0.5251	positive
Leptobacillium	*Tricholoma*	0.5563	positive
Leptobacillium	*Fusicolla*	0.6341	positive
Leptobacillium	*Thermoascus*	0.5416	positive
Leptobacillium	*Arthrographis*	0.615	positive
Leptobacillium	*Paraphoma*	0.7176	positive
Leptobacillium	*Remersonia*	0.5824	positive
Leptobacillium	*Exophiala*	0.5398	positive
Leptobacillium	*Colletotrichum*	0.6343	positive
Arxiella	*Cystobasidium*	0.6797	negative
Arxiella	*Gibellulopsis*	0.5075	positive
Arxiella	*Rhodotorula*	0.6065	negative
Arxiella	*Botryotrichum*	0.6031	positive
Arxiella	*Pyrenochaetopsis*	0.5732	positive
Arxiella	*Candida*	0.6747	positive
Arxiella	*Gymnoascus*	0.7339	positive

Arxiella	*Stolonocarpus*	0.7143	positive
Arxiella	*Cercospora*	0.5497	positive
Arxiella	*Myrmecridium*	0.5583	positive
Arxiella	*Wallemia*	0.5075	positive
Arxiella	*Mycothermus*	0.6198	positive
Arxiella	*Lepiota*	0.8713	positive
Arxiella	*Fusicolla*	0.5534	positive
Arxiella	*Thermoascus*	0.5228	positive
Arxiella	*Arthrographis*	0.6545	positive
Arxiella	*Paraphoma*	0.5613	positive
Arxiella	*Ceratobasidium*	0.5459	positive
Arxiella	*Remersonia*	0.5075	positive
Arxiella	*Xerochrysium*	0.5504	positive
Arxiella	*Mycoarthris*	0.6273	positive
Arxiella	*Exophiala*	0.6378	positive
Arxiella	*Tomentella*	0.4993	positive
Arxiella	*Colletotrichum*	0.7051	positive
Cystobasidium	*Metarhizium*	0.5622	negative
Cystobasidium	*Gymnoascus*	0.5916	negative
Cystobasidium	*Lepiota*	0.6155	negative
Cystobasidium	*Arthrographis*	0.5034	negative
Cystobasidium	*Tomentella*	0.5367	negative
Cystobasidium	*Colletotrichum*	0.592	negative
Gibellulopsis	*Botryotrichum*	0.5588	positive
Gibellulopsis	*Rhinocladiella*	0.5168	negative
Gibellulopsis	*Oidiodendron*	0.543	positive
Gibellulopsis	*Cercospora*	0.6034	positive
Gibellulopsis	*Mycothermus*	0.59	positive
Gibellulopsis	*Fusicolla*	0.6148	positive
Gibellulopsis	*Ceratobasidium*	0.683	positive
Gibellulopsis	*Remersonia*	0.6088	positive
Gibellulopsis	*Mycoarthris*	0.5314	positive
Rhodotorula	*Pyrenochaetopsis*	0.5872	negative
Rhodotorula	*Candida*	0.5092	negative
Rhodotorula	*Lepiota*	0.6539	negative
Rhodotorula	*Fusicolla*	0.5589	negative
Rhodotorula	*Xerochrysium*	0.4982	negative
Botryotrichum	*Rhinocladiella*	0.5089	negative
Botryotrichum	*Thelebolus*	0.6196	negative
Botryotrichum	*Gymnoascus*	0.7185	positive
Botryotrichum	*Stolonocarpus*	0.7897	positive
Botryotrichum	*Cercospora*	0.5445	positive
Botryotrichum	*Myrmecridium*	0.7324	positive
Botryotrichum	*Clonostachys*	0.6118	positive

Botryotrichum	*Stachybotrys*	0.5103	positive
Botryotrichum	*Thermoascus*	0.652	positive
Botryotrichum	*Ceratobasidium*	0.7585	positive
Botryotrichum	*Remersonia*	0.5529	positive
Botryotrichum	*Xerochrysium*	0.6134	positive
Botryotrichum	*Tomentella*	0.56	positive
Botryotrichum	*Colletotrichum*	0.7403	positive
Pyrenochaetopsis	*Candida*	0.7	positive
Pyrenochaetopsis	*Lectera*	0.5265	positive
Pyrenochaetopsis	*Lepiota*	0.5841	positive
Rhinocladiella	*Lectera*	0.5733	negative
Rhinocladiella	*Stolonocarpus*	0.5056	negative
Rhinocladiella	*Leohumicola*	0.5671	negative
Rhinocladiella	*Clonostachys*	0.5435	negative
Rhinocladiella	*Fusicolla*	0.6227	negative
Rhinocladiella	*Remersonia*	0.6362	negative
Thelebolus	*Gymnoascus*	0.5278	negative
Thelebolus	*Clonostachys*	0.6107	negative
Metarhizium	*Candida*	0.7382	positive
Metarhizium	*Curvularia*	0.5239	negative
Metarhizium	*Lectera*	0.7088	positive
Metarhizium	*Leohumicola*	0.5882	positive
Metarhizium	*Myrmecridium*	0.5176	positive
Metarhizium	*Echria*	0.6471	positive
Metarhizium	*Clonostachys*	0.5765	positive
Metarhizium	*Lepiota*	0.5133	positive
Metarhizium	*Tricholoma*	0.5563	positive
Metarhizium	*Kazachstania*	0.5121	positive
Metarhizium	*Fusicolla*	0.6563	positive
Metarhizium	*Thermoascus*	0.5224	positive
Metarhizium	*Microdochium*	0.6676	positive
Metarhizium	*Arthrographis*	0.5165	positive
Metarhizium	*Paraphoma*	0.6824	positive
Metarhizium	*Remersonia*	0.7706	positive
Metarhizium	*Exophiala*	0.6077	positive
Metarhizium	*Paramyrothecium*	0.55	positive
Metarhizium	*Tomentella*	0.7111	positive
Metarhizium	*Colletotrichum*	0.5901	positive
Candida	*Lectera*	0.8618	positive
Candida	*Leohumicola*	0.7265	positive
Candida	*Echria*	0.5912	positive
Candida	*Clonostachys*	0.6059	positive
Candida	*Lepiota*	0.7286	positive
Candida	*Tricholoma*	0.6078	positive

Candida	*Kazachstania*	0.5313	positive
Candida	*Fusicolla*	0.5408	positive
Candida	*Microdochium*	0.7412	positive
Candida	*Arthrographis*	0.6926	positive
Candida	*Paraphoma*	0.6618	positive
Candida	*Remersonia*	0.5735	positive
Candida	*Exophiala*	0.7699	positive
Candida	*Tomentella*	0.5719	positive
Curvularia	*Gymnoascus*	0.5263	negative
Curvularia	*Myrmecridium*	0.5504	negative
Curvularia	*Lepiota*	0.5491	negative
Curvularia	*Arthrographis*	0.6289	negative
Epicoccum	*Tricholoma*	0.5121	negative
Lectera	*Leohumicola*	0.8529	positive
Lectera	*Echria*	0.7794	positive
Lectera	*Clonostachys*	0.7265	positive
Lectera	*Tricholoma*	0.5887	positive
Lectera	*Microdochium*	0.8588	positive
Lectera	*Arthrographis*	0.5135	positive
Lectera	*Remersonia*	0.6088	positive
Lectera	*Exophiala*	0.6401	positive
Lectera	*Tomentella*	0.5615	positive
Oidiodendron	*Cercospora*	0.525	positive
Oidiodendron	*Mycothermus*	0.7793	positive
Oidiodendron	*Stachybotrys*	0.6554	positive
Oidiodendron	*Mycoarthris*	0.5751	positive
Oidiodendron	*Exophiala*	0.6406	positive
Oidiodendron	*Tomentella*	0.5567	positive
Gymnoascus	*Stolonocarpus*	0.8261	positive
Gymnoascus	*Myrmecridium*	0.7941	positive
Gymnoascus	*Clonostachys*	0.6371	positive
Gymnoascus	*Lepiota*	0.7177	positive
Gymnoascus	*Kazachstania*	0.5315	positive
Gymnoascus	*Thermoascus*	0.7658	positive
Gymnoascus	*Arthrographis*	0.6376	positive
Gymnoascus	*Ceratobasidium*	0.5351	positive
Gymnoascus	*Exophiala*	0.4993	positive
Gymnoascus	*Tomentella*	0.5948	positive
Gymnoascus	*Colletotrichum*	0.8021	positive
Stolonocarpus	*Leohumicola*	0.5526	positive
Stolonocarpus	*Myrmecridium*	0.8311	positive
Stolonocarpus	*Thermomyces*	0.5022	positive
Stolonocarpus	*Clonostachys*	0.5748	positive
Stolonocarpus	*Lepiota*	0.6687	positive

Stolonocarpus	*Fusicolla*	0.6119	positive
Stolonocarpus	*Thermoascus*	0.6516	positive
Stolonocarpus	*Arthrographis*	0.6466	positive
Stolonocarpus	*Ceratobasidium*	0.5985	positive
Stolonocarpus	*Remersonia*	0.5956	positive
Stolonocarpus	*Xerochrysium*	0.5963	positive
Stolonocarpus	*Tomentella*	0.5851	positive
Stolonocarpus	*Colletotrichum*	0.8658	positive
Leohumicola	*Myrmecridium*	0.5618	positive
Leohumicola	*Echria*	0.5882	positive
Leohumicola	*Clonostachys*	0.6324	positive
Leohumicola	*Tricholoma*	0.6387	positive
Leohumicola	*Fusicolla*	0.563	positive
Leohumicola	*Microdochium*	0.8118	positive
Leohumicola	*Remersonia*	0.5529	positive
Leohumicola	*Exophiala*	0.5044	positive
Cercospora	*Mycothermus*	0.7469	positive
Cercospora	*Arthrographis*	0.5094	positive
Cercospora	*Ceratobasidium*	0.5159	positive
Cercospora	*Mycoarthris*	0.5258	positive
Myrmecridium	*Thermomyces*	0.5118	positive
Myrmecridium	*Clonostachys*	0.7529	positive
Myrmecridium	*Lepiota*	0.5752	positive
Myrmecridium	*Kazachstania*	0.5975	positive
Myrmecridium	*Thermoascus*	0.8079	positive
Myrmecridium	*Arthrographis*	0.7941	positive
Myrmecridium	*Ceratobasidium*	0.5052	positive
Myrmecridium	*Xerochrysium*	0.5867	positive
Myrmecridium	*Exophiala*	0.5133	positive
Myrmecridium	*Tomentella*	0.6993	positive
Myrmecridium	*Colletotrichum*	0.8197	positive
Thermomyces	*Lepiota*	0.5162	positive
Thermomyces	*Arthrographis*	0.5314	positive
Thermomyces	*Xerochrysium*	0.6519	positive
Thermomyces	*Tomentella*	0.6282	positive
Thermomyces	*Colletotrichum*	0.5327	positive
Wallemia	*Paraphoma*	0.7235	positive
Echria	*Tricholoma*	0.5269	positive
Echria	*Microdochium*	0.8412	positive
Echria	*Paraphoma*	0.5	positive
Echria	*Remersonia*	0.5147	positive
Mycothermus	*Lepiota*	0.5976	positive
Mycothermus	*Kazachstania*	0.6317	positive
Mycothermus	*Stachybotrys*	0.6716	positive

Mycothermus	*Arthrographis*	0.536	positive
Mycothermus	*Ceratobasidium*	0.5721	positive
Mycothermus	*Mycoarthris*	0.7396	positive
Mycothermus	*Exophiala*	0.6154	positive
Mycothermus	*Tomentella*	0.5602	positive
Clonostachys	*Lepiota*	0.5664	positive
Clonostachys	*Kazachstania*	0.5445	positive
Clonostachys	*Thermoascus*	0.5666	positive
Clonostachys	*Arthrographis*	0.6956	positive
Clonostachys	*Remersonia*	0.7029	positive
Clonostachys	*Xerochrysium*	0.5185	positive
Clonostachys	*Exophiala*	0.5546	positive
Clonostachys	*Tomentella*	0.6993	positive
Clonostachys	*Colletotrichum*	0.5548	positive
Lepiota	*Kazachstania*	0.6037	positive
Lepiota	*Fusicolla*	0.5007	positive
Lepiota	*Arthrographis*	0.7276	positive
Lepiota	*Ceratobasidium*	0.5126	positive
Lepiota	*Xerochrysium*	0.6137	positive
Lepiota	*Mycoarthris*	0.6168	positive
Lepiota	*Exophiala*	0.7574	positive
Lepiota	*Tomentella*	0.6211	positive
Lepiota	*Colletotrichum*	0.6214	positive
Tricholoma	*Kazachstania*	0.7489	positive
Tricholoma	*Thermoascus*	0.5545	positive
Tricholoma	*Microdochium*	0.5931	positive
Tricholoma	*Arthrographis*	0.5169	positive
Tricholoma	*Exophiala*	0.5771	positive
Tricholoma	*Tomentella*	0.5389	positive
Kazachstania	*Stachybotrys*	0.6199	positive
Kazachstania	*Thermoascus*	0.7135	positive
Kazachstania	*Arthrographis*	0.714	positive
Kazachstania	*Remersonia*	0.4989	positive
Kazachstania	*Xerochrysium*	0.6368	positive
Kazachstania	*Exophiala*	0.6908	positive
Kazachstania	*Tomentella*	0.7858	positive
Kazachstania	*Colletotrichum*	0.6222	positive
Fusicolla	*Paraphoma*	0.5408	positive
Fusicolla	*Ceratobasidium*	0.5254	positive
Fusicolla	*Remersonia*	0.6726	positive
Fusicolla	*Iodophanus*	0.5508	positive
Fusicolla	*Colletotrichum*	0.5404	positive
Stachybotrys	*Ceratobasidium*	0.7251	positive
Stachybotrys	*Xerochrysium*	0.575	positive

Stachybotrys	*Mycoarthris*	0.6827	positive
Stachybotrys	*Exophiala*	0.6391	positive
Stachybotrys	*Tomentella*	0.6315	positive
Stachybotrys	*Colletotrichum*	0.4989	positive
Thermoascus	*Arthrographis*	0.6184	positive
Thermoascus	*Xerochrysium*	0.5145	positive
Thermoascus	*Exophiala*	0.5816	positive
Thermoascus	*Tomentella*	0.7472	positive
Thermoascus	*Colletotrichum*	0.8137	positive
Microdochium	*Remersonia*	0.5529	positive
Arthrographis	*Xerochrysium*	0.6496	positive
Arthrographis	*Mycoarthris*	0.5364	positive
Arthrographis	*Exophiala*	0.5929	positive
Arthrographis	*Tomentella*	0.6617	positive
Arthrographis	*Colletotrichum*	0.6513	positive
Paraphoma	*Coprinellus*	0.4985	positive
Paraphoma	*Paramyrothecium*	0.5	positive
Ceratobasidium	*Mycoarthris*	0.7399	positive
Ceratobasidium	*Colletotrichum*	0.5167	positive
Remersonia	*Xerochrysium*	0.5185	positive
Remersonia	*Tomentella*	0.6711	positive
Remersonia	*Colletotrichum*	0.6034	positive
Xerochrysium	*Mycoarthris*	0.5188	positive
Xerochrysium	*Exophiala*	0.5201	positive
Xerochrysium	*Tomentella*	0.744	positive
Xerochrysium	*Colletotrichum*	0.7391	positive
Exophiala	*Tomentella*	0.7846	positive
Exophiala	*Colletotrichum*	0.4989	positive
Tomentella	*Colletotrichum*	0.765	positive
